# Genotyping of *Mycoplasma pneumoniae* strains isolated in Japan during 2019 and 2020: spread of *p1* gene type 2c and 2j variant strains

**DOI:** 10.3389/fmicb.2023.1202357

**Published:** 2023-06-19

**Authors:** Tsuyoshi Kenri, Tsutomu Yamazaki, Hitomi Ohya, Michio Jinnai, Yoichiro Oda, Sadasaburo Asai, Rikako Sato, Nobuhisa Ishiguro, Tomohiro Oishi, Atsuko Horino, Hiroyuki Fujii, Toru Hashimoto, Hiroshi Nakajima, Keigo Shibayama

**Affiliations:** ^1^Department of Bacteriology II, National Institute of Infectious Diseases, Tokyo, Japan; ^2^Wakaba Children’s Clinic, Saitama, Japan; ^3^Kanagawa Prefectural Institute of Public Health, Kanagawa, Japan; ^4^Chigasaki Municipal Hospital, Kanagawa, Japan; ^5^Asai Children’s Clinic, Osaka, Japan; ^6^Department of Pediatrics, Hokkaido University Graduate School of Medicine, Sapporo, Japan; ^7^Department of Clinical Infectious Diseases, Kawasaki Medical School, Okayama, Japan; ^8^Kurashiki Central Hospital, Okayama, Japan; ^9^Okayama Prefectural Institute for Environmental Science and Public Health, Okayama, Japan; ^10^Department of Bacteriology, Nagoya University Graduate School of Medicine, Nagoya, Japan

**Keywords:** *Mycoplasma pneumoniae*, *Mycoplasmoides pneumoniae*, whole-genome single-nucleotide polymorphism, infectious diseases surveillance, macrolide resistance, antigenic variation, protective immunity, *p1* gene genotyping

## Abstract

We characterized 118 *Mycoplasma pneumoniae* strains isolated from three areas of Japan (Saitama, Kanagawa, and Osaka) during the period of 2019 and 2020. Genotyping of the *p1* gene in these strains revealed that 29 of them were type 1 lineage (29/118, 24.6%), while 89 were type 2 lineage (89/118, 75.4%), thereby indicating that type 2 lineage was dominant in this period. The most prevalent variant of type 2 lineage was type 2c (57/89, 64%), while the second-most was type 2j, a novel variant identified in this study (30/89, 33.7%). Type 2j *p1* is similar to type 2 g *p1*, but cannot be distinguished from reference type 2 (classical type 2) using the standard polymerase chain reaction-restriction fragment length polymorphism analysis (PCR-RFLP) with *Hae*III digestion. Thus, we used *Mbo*I digestion in the PCR-RFLP analysis and re-examined the data from previous genotyping studies as well. This revealed that most strains reported as classical type 2 after 2010 in our studies were actually type 2j. The revised genotyping data showed that the type 2c and 2j strains have been spreading in recent years and were the most prevalent variants in Japan during the time-period of 2019 and 2020. We also analyzed the macrolide-resistance (MR) mutations in the 118 strains. MR mutations in the 23S rRNA gene were detected in 29 of these strains (29/118, 24.6%). The MR rate of type 1 lineage (14/29, 48.3%) was still higher than that of type 2 lineage (15/89, 16.9%); however, the MR rate of type 1 lineage was lower than that found in previous reports published in the 2010s, while that of type 2 lineage strains was slightly higher. Thus, there is a need for continuous surveillance of the *p1* genotype and MR rate of *M. pneumoniae* clinical strains, to better understand the epidemiology and variant evolution of this pathogen, although *M. pneumoniae* pneumonia cases have decreased significantly since the COVID-19 pandemic.

## 1. Introduction

*Mycoplasma pneumoniae* (*Mycoplasmoides pneumoniae*) is a cell wall-lacking bacterium that is a common cause of pneumonia and bronchitis in humans. *M. pneumoniae* clinical isolates can be classified into two distinct genetic groups: type 1 and 2 lineages (Xiao et al., 2015; Diaz et al., 2017; Lee et al., 2019; Kenri et al., 2020). Standard genotyping methods developed for *M. pneumoniae*, including multi-locus variable-number tandem repeat analysis (MLVA; Degrange et al., 2009), multi-locus sequence typing (MLST; Brown et al., 2015), and single nucleotide polymorphism (SNP) analysis (Touati et al., 2015; Zhao et al., 2021), can easily discriminate between these two lineages (Supplementary Figure S1). The classical reference strains of the type 1 and 2 lineages are M129 and FH, respectively (GenBank accession nos. U00089 and CP010546, respectively; Lipman et al., 1969; Barile et al., 1988; Himmelreich et al., 1996). The detection rate of type 1 and 2 lineage strains in clinical specimens fluctuates depending on the area and time-point of genotyping research (Pereyre et al., 2007; Dumke et al., 2010; Diaz et al., 2015; Zhao et al., 2019). In Japan, several epidemiological studies have observed that type 1 and 2 lineages alternately become dominant, in cycles of about 10 years (Sasaki et al., 1996; Kenri et al., 2008, 2020). Between the type 1 and 2 lineages, the *p1* (MPN141) and *orf6* (MPN142) genes also exhibit sequence polymorphism (Su et al., 1990; Ruland et al., 1994). The *p1* and *orf6* genes encode the P1 and P40/P90 proteins, respectively, which are components of the adhesin protein complex (nap) and responsible for the infection process and pathogenesis of this bacterium (Waites et al., 2017; Vizarraga et al., 2020). The sequence polymorphism of *p1* and *orf6* can be discriminated using PCR-restriction fragment length polymorphism (RFLP) analysis (Cousin-Allery et al., 2000) or sequencing of the *p1* operon. These analyses enable further classification of *p1* and *orf6* genes, in addition to that of the classical type 1 and 2 sequences of the M129 and FH strains. Several variants of *p1* and *orf6* in the type 1 and 2 lineages are known to date (Kenri et al., 2020; Xiao et al., 2020). These *p1* and *orf6* variants are generated by DNA recombination between repetitive sequences (RepMP elements) in the *M. pneumoniae* genome (Kenri et al., 1999; Hakim et al., 2021). Variations in the *p1* and *orf6* genes cause amino acid substitutions in the P1 and P40/P90 proteins, respectively, and may affect the antigenicity and infectivity of this bacterium, as well as the host immune response. Therefore, it is important to undertake surveillance of new *p1* and *orf6* gene variants.

Macrolides are first-line drugs for the clinical treatment of *M. pneumoniae* pneumonia (Yamazaki and Kenri, 2016). However, macrolide-resistant *M. pneumoniae* (MRMP) strains have spread since the 2000s, especially in East Asian countries (Meyer Sauteur et al., 2016; Pereyre et al., 2016; Kim et al., 2022), raising concerns regarding the treatment of *M. pneumoniae* infections. An interesting epidemiological feature of recent MRMP strain profiles is the high macrolide-resistance (MR) rate of type 1 lineage strains, as compared to that of type 2 strains. Most type 1 clinical isolates were MRMPs in the early 2010s, whereas type 2 lineage strains were largely macrolide-susceptible *M. pneumoniae* (MSMP; Liu et al., 2009; Zhao et al., 2019; Kenri et al., 2020; Ishiguro et al., 2021; Nakamura et al., 2021). This may be because of the excessive clinical use of macrolides in the 2000s, when type 1 lineage strains were dominant. Since the late 2010s, type 2 lineage strains have become clinically prevalent in Japan, and most of these type 2 lineage strains are MSMP. However, in China and South Korea, a gradual increase in the MR rate of the type 2 lineage has been reported in recent years (Wang et al., 2021; Guo et al., 2022; Lee et al., 2022; Wang et al., 2022). Therefore, it is particularly important to monitor the MR rate of the type 2 lineage strains in Japan. In this study, we analyzed the *p1* gene genotype and MR mutations in 118 *M. pneumoniae* strains isolated in Japan during the period of 2019 and 2020.

## 2. Materials and methods

### 2.1. *Mycoplasma pneumoniae* isolates

The 118 *M. pneumoniae* strains analyzed in this study are listed in Supplementary Table S1. These strains were isolated from throat swab and sputum specimens by means of culture in PPLO (pleuropneumonia-like organisms) medium, as reported previously (Kenri et al., 2008, 2020). Throat swabs and sputa were collected from patients with acute respiratory infections, between August 2019 and April 2020, from three areas of Japan (Saitama, Kanagawa, and Osaka prefectures). Swabs were collected at the Wakaba Children’s Clinic in Saitama, Chigasaki Municipal Hospital and sentinel hospitals for infectious diseases surveillance in Kanagawa, and the Asai Children’s Clinic in Osaka. Sputa were collected at Kishiwada Tokushukai Hospital in Osaka. The throat swabs and sputa were collected and analyzed under the approval of the ethics committees of the National Institute of Infectious Diseases (No. 1487) and the Kanagawa Prefectural Institute of Public Health (No. R2-1).

### 2.2. *p1* typing and detection of MR mutations

**Figure 1:**
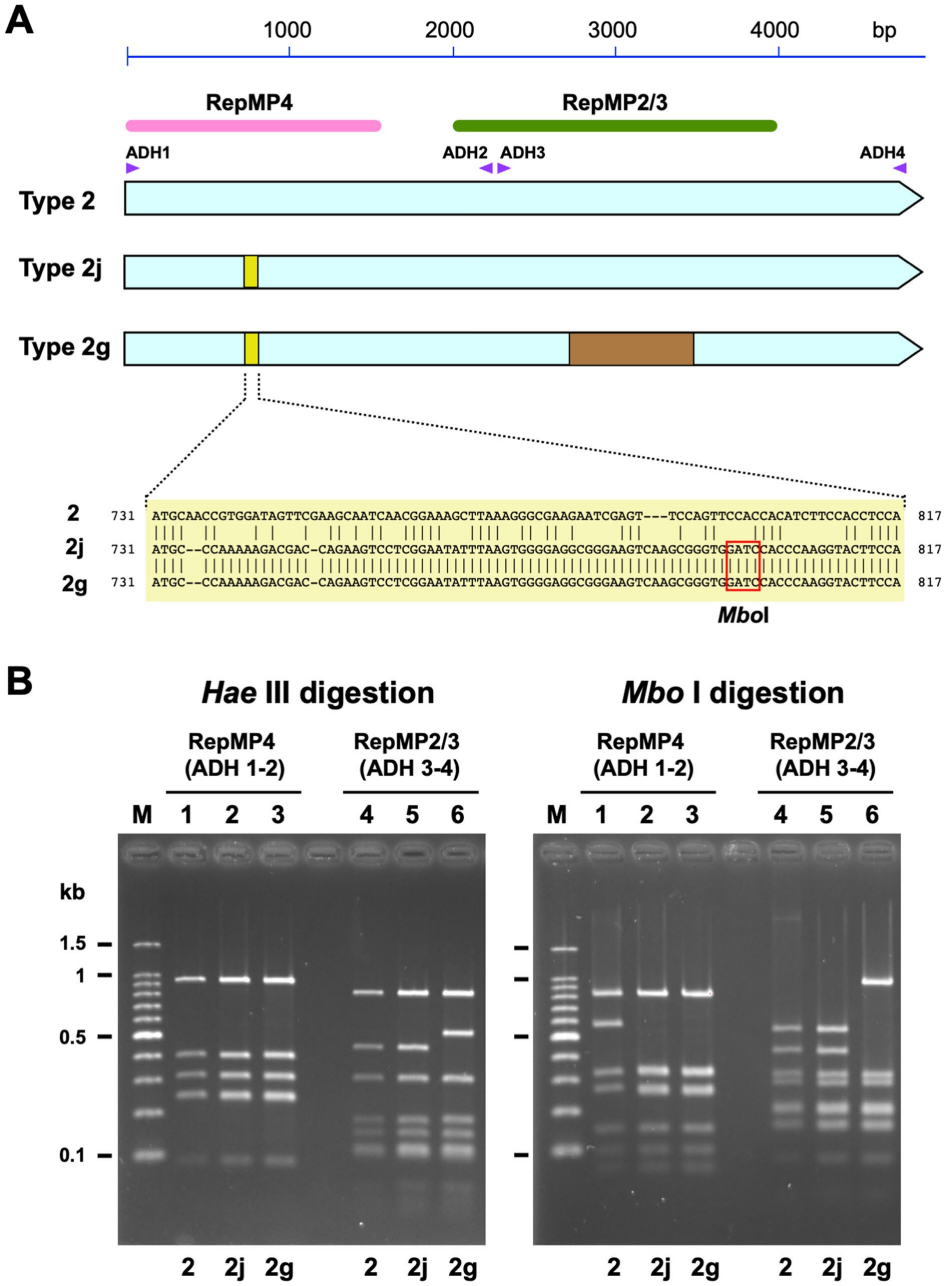
Discrimination of type 2 lineage *p1* genes using PCR-RFLP analysis. **(A)** A schematic illustration of type 2 *p1* gene variants. The three light blue arrows indicate type 2, 2j, and 2 g genes. The small yellow boxes indicate the location of variation in the type 2j and 2 g *p1* genes. The sequences of the variations are shown below. Type 2j and 2 g *p1* have an *Mbo*I site in this region. The brown rectangle indicates the location of the variation in the type 2 g *p1* gene. The pink and green bars indicate the locations of the RepMP4 and RepMP2/3 elements, respectively. The small purple triangles indicate the positions of ADH1, ADH2, ADH3, and ADH4 primers used for PCR-RFLP analysis. **(B)** Band patterns obtained for PCR-RFLP analysis of type 2, 2j, and 2 g *p1* genes upon 2% agarose-gel electrophoresis. The left and the right panels show the *Hae*III and *Mbo*I digestion patterns of the same samples, respectively. The RepMP4 region (ADH1-2 amplicon) of the FH (type 2), Y12-38 (type 2j), and K708 (type 2 g) strains have been analyzed in lanes 1, 2, and 3, respectively. The RepMP2/3 region (ADH3-4 amplicon) of the same strains has been analyzed in lanes 4, 5, and 6, respectively. Lane M shows the 100 bp ladder DNA size marker.

*p1* genotyping of *M. pneumoniae* isolates was performed using PCR-RFLP analysis, as reported previously (Cousin-Allery et al., 2000). Briefly, *p1* gene sequences containing the RepMP4 or RepMP2/3 regions were amplified using PCR, with the ADH1 (5′-CTGCCTTGTCCAAGTCCACT-3′) and ADH2 (5′-AACCTTG TCGGGAAGAGCTG-3′) or ADH3 (5′-CGAGTTTGCTGCTAAC GAGT-3′) and ADH4 (5′-CTTGACTGATACCTGTGCGG-3′) primer sets (Figure 1A). The amplified fragments were digested using *Hae*III or *Mbo*I restriction enzymes (Takara Bio, Shiga, Japan). To visualize the RFLP patterns for typing, digested fragments were analyzed using 2% agarose-gel electrophoresis. To sequence the *p1* operon, a previously reported method and primer set were used (Katsukawa et al., 2019). The MR of the *M. pneumoniae* isolates was examined by detecting MR mutations in the 23S rRNA gene, using a previously reported method (Matsuoka et al., 2004).

### 2.3. Genome sequencing and whole-genome single-nucleotide polymorphism (WG-SNP) analysis

Six *M. pneumoniae* strains KPI-025, Y12-4, Y12-24, Y12-38, OA-57, and OA-63 were sequenced in this study (Supplementary Figures S1; Supplementary Table S1). The strains were cultured in 10 mL of PPLO broth, following which the cells were harvested. Genomic DNA was extracted using the QIAamp DNA Mini Kit (Qiagen, Hilden, Germany). Libraries of 500 bp-long genomic DNA inserts were prepared using the Illumina DNA Prep (M) Tagmentation Kit (Illumina, San Diego, CA, United States). Next-generation sequencing was performed at the Genome-Lead Corporation (Kagawa, Japan), using the Illumina MiSeq platform and MiSeq Reagent Micro Kit v2 (Illumina). The obtained paired-end reads were assembled *de novo* using Shovill v1.1.0.,^1^ with default parameters. WG-SNP analysis was performed using the CSI Phylogeny 1.4 pipeline^2^ (Kaas et al., 2014). The genome sequences of the type 1 strain M129-B7 (GenBank accession no. CP003913) and type 2 strain FH (GenBank accession no. CP017327) were used as reference sequences for the analyses of type 1 and type 2 lineage strains, respectively. Phylogenetic trees were visualized using FigTree v1.4.4 software.^3^

### 2.4. Phylogenetic analysis of type 1 lineage strains

MPN295, MPN607, and MPN678 gene fragments were amplified from type 1 *M. pneumoniae* genomic DNA using PCR, with the primer sets listed in Table 1. PCR was performed in multiplex or separately for the three genes. In the multiplex PCR conditions, 4-times higher amounts of the MPN295 primer set than those of the MPN607 and MPN678 primer sets were used in the PCR reaction mixture. The PCR products of the MPN295, MPN607, and MPN678 genes were treated with the restriction enzyme *Apo*I (New England Biolabs, Ipswich, MA, United States) and then analyzed using 2% agarose-gel electrophoresis, to visualize the digestion pattern (see Section 3.3).

**Table 1:** PCR primers used for the phylogenetic analysis of type 1 lineage strains.

Primers	Sequence	Amplicon size (bp)
MPN295-F	TTGATTGAATTACTTACCTCAA	150
MPN295-R	TAGTGAACACGCCATAAACA	
MPN607-F	CAGTTAATTACGCAAAAGTTTAG	300
MPN607-R	CAAGGTTAAAGACGAGAAGC	
MPN678-F	GAGGACACTGACACTGAGCG	624
MPN678-R	GCCACTCTTGTCGACTATCAC	

### 2.5. Nucleotide sequence accession numbers

The nucleotide sequences of the type 2j *p1* gene operon of the Y4-20, KT19, and OA-29 strains were deposited in the DDBJ/ENA/GenBank databases, under the accession numbers LC588412, LC588413, and LC588414, respectively. The nucleotide sequence of the type 2b2 *p1* operon of the Y12-24 strain was deposited under the accession number LC753471. Genome sequence data of six *M. pneumoniae* strains, KPI-025, Y12-4, Y12-24, Y12-38, OA-57, and OA-63, were deposited under the accession numbers BSFT01000000, BSFU01000000, BSFV01000000, BSFW01000000, BSFX01000000, and BSFY01000000, respectively.

## 3. Results

### 3.1. Identification of a novel *P1* gene variant 2j during *P1* genotyping and MR analysis of *Mycoplasma pneumoniae* isolates collected during the period of 2019 and 2020

We isolated 118 *M. pneumoniae* strains from patients with acute respiratory infections between August 2019 and April 2020 using a culture method. Throat swab and sputum specimens for *M. pneumoniae* isolation were collected from three areas in Japan (Saitama, Kanagawa, and Osaka prefectures; see Section 2; Supplementary Table S1). We analyzed the *p1* gene genotype and MR mutations in these isolates. *P1* genotyping was performed using standard PCR-RFLP analysis, and several specimens were inspected by means of DNA sequencing. During this sequence inspection, we identified a novel *p1* gene variant, which had a minor sequence change in the RepMP4 region of the *p1* gene, as compared to that of the classical type 2 strains (Figure 1). Although this RepMP4 variation was identical to that of the type 2 g strain reported previously (Katsukawa et al., 2019), the new variant did not exhibit variation in the RepMP2/3 region, unlike that of type 2 g (Figure 1A). We designated this novel variant as type 2j, to distinguish it from classical type 2 and 2 g. Type 2j cannot be discriminated from the classical type 2 using PCR-RFLP typing with *Hae*III digestion, because the sequence change in the RepMP4 region of type 2j and 2 g is not recognized by *Hae*III (Figure 1B, lanes 1–3). Therefore, to distinguish type 2j from classical type 2, we employed *Mbo*I digestion in the PCR-RFLP genotyping, in addition to *Hae*III digestion (Figure 1).

**Figure 2:**
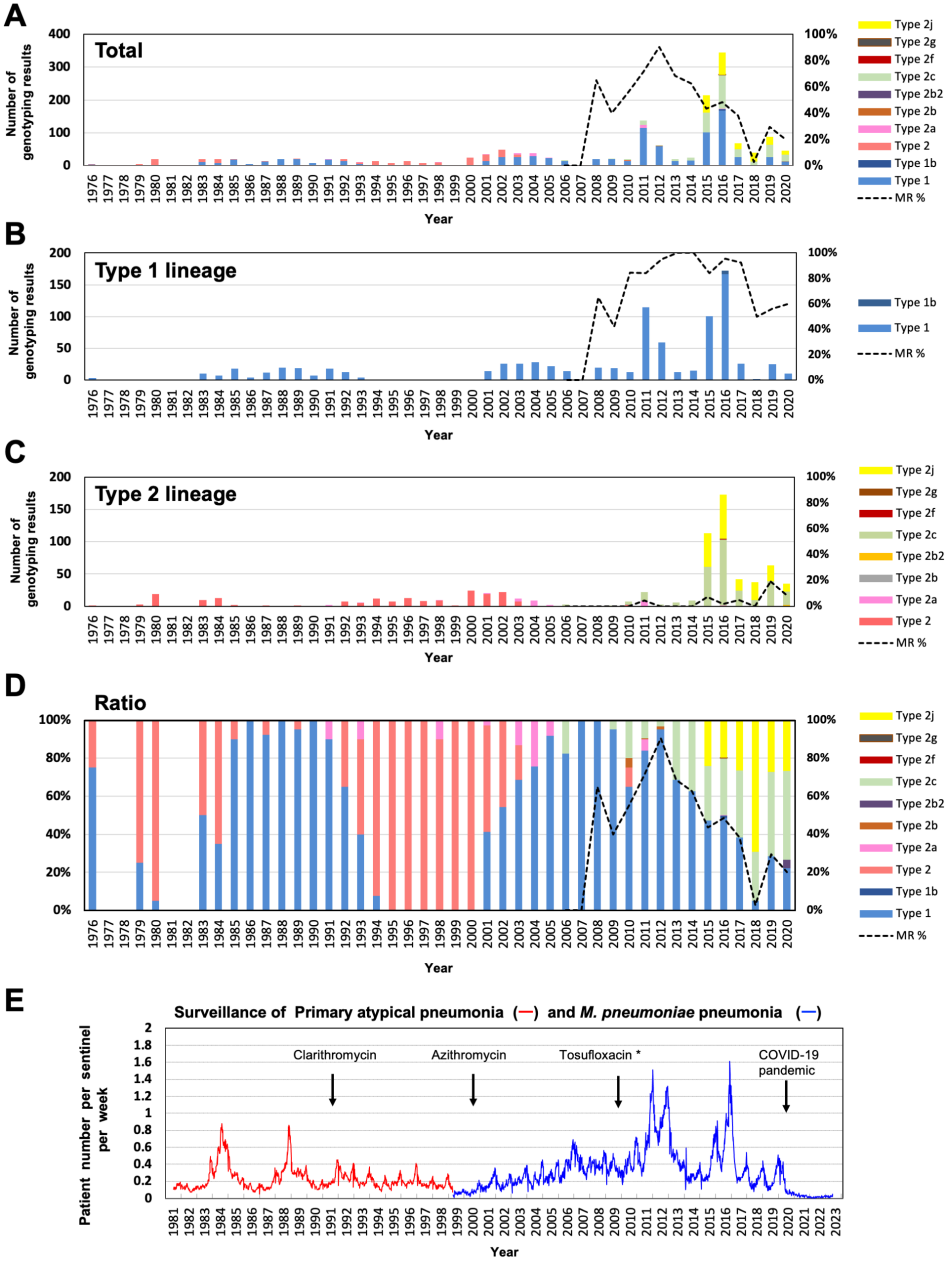
Summary of the *p1* genotyping and macrolide resistance (MR) analysis of the 118 *Mycoplasma pneumoniae* strains isolated in this study. **(A)** Results of *p1* genotyping and MR analysis of the 118 strains isolated in 2019 and 2020. The results have also been represented in the form of graphs. **(B)** Analysis results shown by collection area of the strains. ^*^Two type 1 strains isolated in Kanagawa in 2019 and 2020 carry a A2064G MR mutation, and one type 2j strain isolated in Osaka hospital in 2019 carries a A2063T MR mutation in the 23S rRNA gene (see Supplementary Table S1). Type 2b2 has also been referred to as type 2e or type 2bv in the reports of other groups (Gullsby et al., 2019; Xiao et al., 2020).

The results of *p1* genotyping and MR mutation analysis are shown in Figure 2. Of the 118 isolates, 29 were *p1* gene type 1, 2 were type 2b2, 57 were type 2c, and 30 were type 2j (Figure 2A). No other variants of the type 1 or 2 lineage were detected, including the type 2 h and 2i reported recently in the United States (Xiao et al., 2020). This result showed that type 2 lineage strains were clinically prevalent in Japan during the time-period of 2019 and 2020 (89/118, 75.4%). Among the type 2 lineages, the type 2c (57/89, 64%) and type 2j (30/89, 33.7%) strains were the most common. To our knowledge, this is also the first time that type 2b2 has been detected in Japan (Figure 2A). The type 2b2 was named variant 2bv or 2e in the previous reports (Gullsby et al., 2019; Xiao et al., 2020).

Macrolide-resistance mutations in the 23S rRNA gene were detected in 29 isolates (29/118, 24.6%). The MR rate of the type 1 isolates was higher (14/29, 48.3%) than that of the type 2 isolates (15/89, 16.9%; Figure 2A). Two type 1 strains (KP3283 and KP3286) derived from Kanagawa carried the A2064G mutation in the 23S rRNA gene, and one type 2j strain (KT-19) derived from Osaka hospital in 2019 carried the A2063T mutation, while the other 26 MR strains carried the A2063G mutation, including two type 2b2 strains (Figure 2B; Supplementary Figure S1).

The proportion of *p1* genotype and MR rate of isolates varied depending on the area (Figure 2B). In the Osaka clinic, the same number of type 1 and type 2 lineage strains were isolated, whereas only type 2 lineage strains were isolated from the Osaka hospital. The MR rate of type 1 strains was higher in the Saitama clinic (66.7%) than that in the Kanagawa (42.9%) and Osaka (43.8%) clinics. In Osaka hospital, the MR rate of the type 2 lineage was higher (40.9%) than that in the other areas (Figure 2B). We think these differences are probably due to locational deference, property of the specimens (sputa or throat swabs), or medical conditions of patients (Supplementary Table S1). It was also reported that MR rate of isolates tended to be higher in larger hospitals (higher order medical institutions) than in clinics (primary medical institutions) in the previous study in Osaka (Katsukawa et al., 2019).

### 3.2. Verification of the past genotyping data of type 2 lineage

The unexpectedly high prevalence of type 2j strains and absence of classical type 2 in the *M. pneumoniae* clinical strains isolated in the time-period of 2019 and 2020 made us wonder whether there were unrecognized type 2j strains in the previous genotyping studies. Thus, we re-analyzed the past clinical isolates. In two previous studies (Katsukawa et al., 2019; Kenri et al., 2020), we reported 172 classical type 2 *M. pneumoniae* isolates between 2010 and August 2019. We re-analyzed the *p1* genes of these strains using PCR-RFLP with *Mbo*I digestion and confirmed that two of these strains in 2010 were classical type 2, whereas the other 170 strains were type 2j (Table 2A). Among these, four draft genome sequenced strains (Y3-12, Y4-15, Y4-20, and Y4-67) from a previous study (Kenri et al., 2020) were also type 2j strains (because complete *p1* gene sequence was not obtained by means of draft genome sequencing, due to the presence of repetitive elements). These four strains clustered with the type 2 g strain K708 in the phylogenetic analysis based on WG-SNP, thereby suggesting a close genetic relationship between the type 2 g and 2j strains (Supplementary Figure S1). The MLVA and MLST types of these type 2j and 2 g strains were 3662 and ST7, respectively.

**Table 2:** Re-analysis of type 2 genotyping data of the past reports.

A								
Year		2010	2015	2016	2017	2018	2019	Total
Number of strains reported as classical type 2 in previous studies^*^		2	52	68	18	27	5	172
Result of re-analysis	Type 2	2	0	0	0	0	0	2
	Type 2j	0	52	68	18	27	5	170

B																					
Year		1976	1979	1980	1983	1984	1985	1987	1992	1993	1994	1995	1996	1997	1998	1999	2000	2001	2002	2003	Total
Number of strains or specimens reported as classical type 2 in previous studies^**^		1	3	19	10	13	2	1	7	5	12	7	13	8	9	1	24	19	22	7	183
Number of strains re-analyzed		1	2	19	9	13	2	1	7	2	3	5	3	3	2	0	0	0	0	6	78
Result of re-analysis	Type 2	1	2	19	9	13	2	1	7	2	3	5	3	3	2	−	−	−	−	6	78
	Type 2j	0	0	0	0	0	0	0	0	0	0	0	0	0	0	−	−	−	−	0	0

^*^These data were reported in Kenri et al. (2020).

^**^These data were reported in Sasaki et al. (1996), Kenri et al. (2008) (also see text, Figure 2; Supplementary Figure S2).

We also reported *p1* genotyping results of the *M. pneumoniae* DNA in clinical specimens using PCR-based typing methods, without isolating bacteria (Ishiguro et al., 2021; Nakamura et al., 2021). In the Hokkaido prefecture, we reported 38 typing results of classical type 2 *p1*, in the specimens collected between 2016 and 2019 (Ishiguro et al., 2021). Of these specimens, 28 were re-analyzed using PCR-RFLP with *Mbo*I digestion and *p1* sequencing, and all specimens were confirmed to be type 2j. In the Okayama prefecture, we reported 24 classical type 2 specimens between 2016 and 2018 (Nakamura et al., 2021). Re-analysis of these specimens confirmed that all *p1* genes were type 2j.

**Figure 3:**
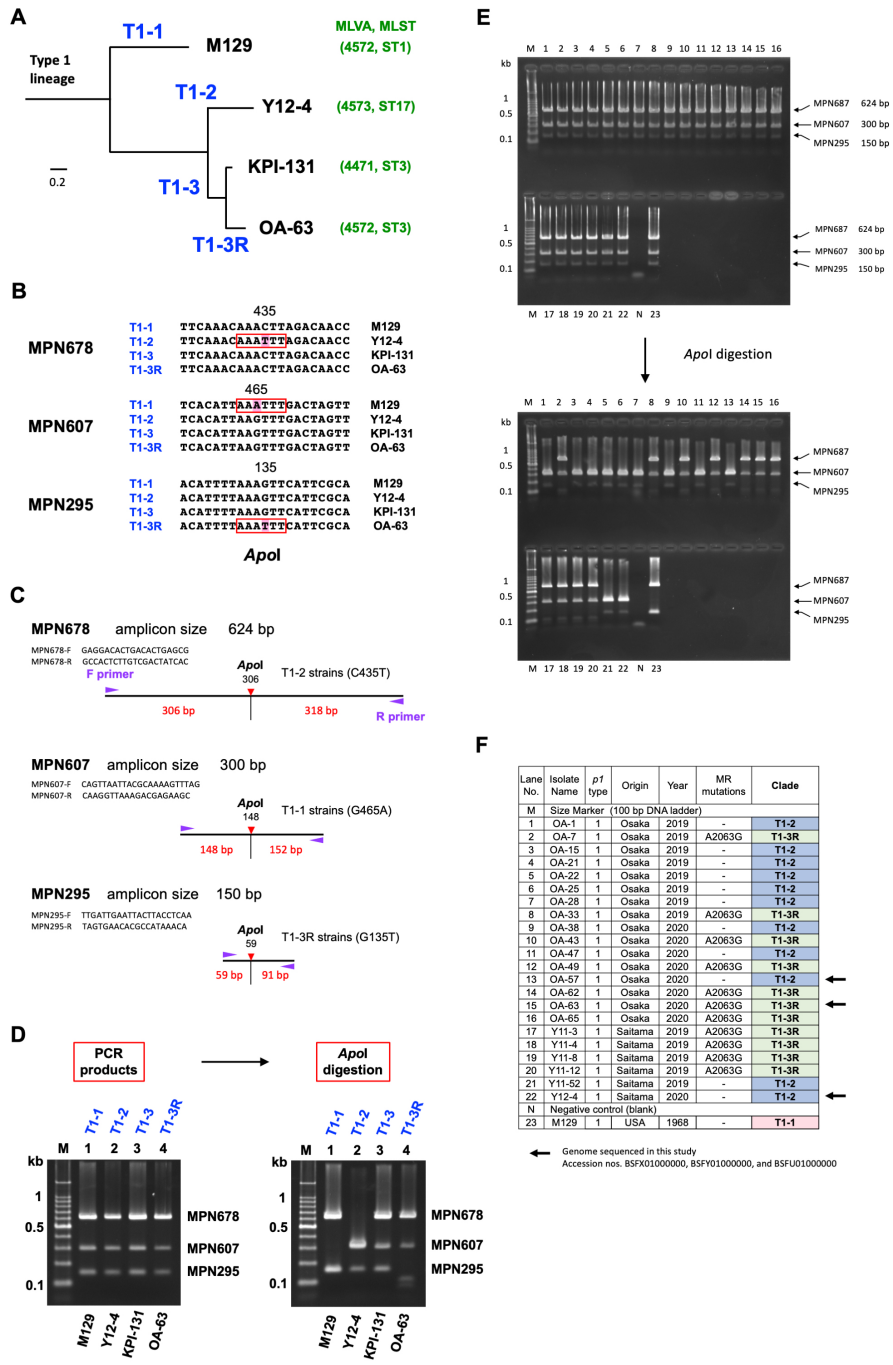
Integrated revised data of *M. pneumoniae* genotyping over the past 40  years. **(A)** Annual typing number and proportion of *p1* gene types. The different *p1* gene variants are shown in different colors. The dotted line shows the MR rate of total isolates after 2006. **(B)** Annual number of type 1 lineage genotyping results. The dotted line shows the MR rate of type 1 lineage isolates after 2006. **(C)** Annual number of type 2 lineage genotyping results. The dotted line shows the MR rate of type 2 lineage isolates after 2006. **(D)** Trend of *p1* gene genotypes (annual ratio of detected genotype). The dotted line shows the MR rate of total isolates after 2006. Detailed data of genotyping and MR analysis are shown in Supplementary Figure S2. **(E)** Surveillance data of *M. pneumoniae* pneumonia in Japan, obtained from the National Epidemiological Surveillance of Infectious Diseases (https://www.niid.go.jp/niid/ja/10/2096-weeklygraph/1659-18myco.html). Surveillance data of primary atypical pneumonia (red line, April 1981–March 1999) and *M. pneumoniae* pneumonia (blue line, after April 1999). The years of introduction of clarithromycin and azithromycin for clinical treatment for *M. pneumoniae* pneumonia in Japan are indicated (Yamazaki and Kenri, 2016). The introduction year of tosufloxacin for children clinical treatments is also indicated. ^*^Tosufloxacin has been officially recommended for pediatric *M. pneumoniae* pneumonia treatment since 2017.

We also re-examined the genotyping results before 2003. We reported 183 classical type 2 *M. pneumoniae* strains and specimens between 1976 and 2003 in previous studies (Sasaki et al., 1996; Kenri et al., 2008). Of these, 78 strains were available for re-analysis and examined using PCR-RFLP with *Mbo*I digestion (Table 2B). In contrast to the re-analysis of strains in the 2010s, no type 2j strain was found among the 78 strains isolated between 1976 and 2003. All 78 strains were classical type 2, suggesting that type 2j strains were not present or were rare before the 2000s (Figure 3; Supplementary Figure S2). This is consistent with lower MR rate of type 2j strains (1.5%, 1/201) compared to that of type 2c (8.1%, 24/295; Supplementary Figure S2). Although when and where the first type 2j strain appeared is unknown, type 2j strains were minor in circulating variants until the middle of 2010s and were not extensively exposed to clinical macrolide treatments in the 2000s.

During this re-analysis of past classical type 2 strains using PCR-RFLP with *Mbo*I, we noticed a single SNP in *p1* gene that affected *Mbo*I digestion in the RepMP2/3 region (ADH3-ADH4 amplicon). Some classical type 2 and type 2b strains carry this SNP in the *p1* gene (T to A transversion at the 2,883 nt position corresponds to the FH *p1* gene). This SNP is a useful marker to discriminate strains belonging to the type 2b branch in the phylogenetic tree of the type 2 lineage (Supplementary Figure S1). Strains carrying this SNP exhibited a PCR-RFLP pattern similar to that of type 2 g in the RepMP2/3 region (Supplementary Figure S3).

### 3.3. Phylogenetic relations between macrolide resistant and susceptible type 1 strains

**Figure 4:**
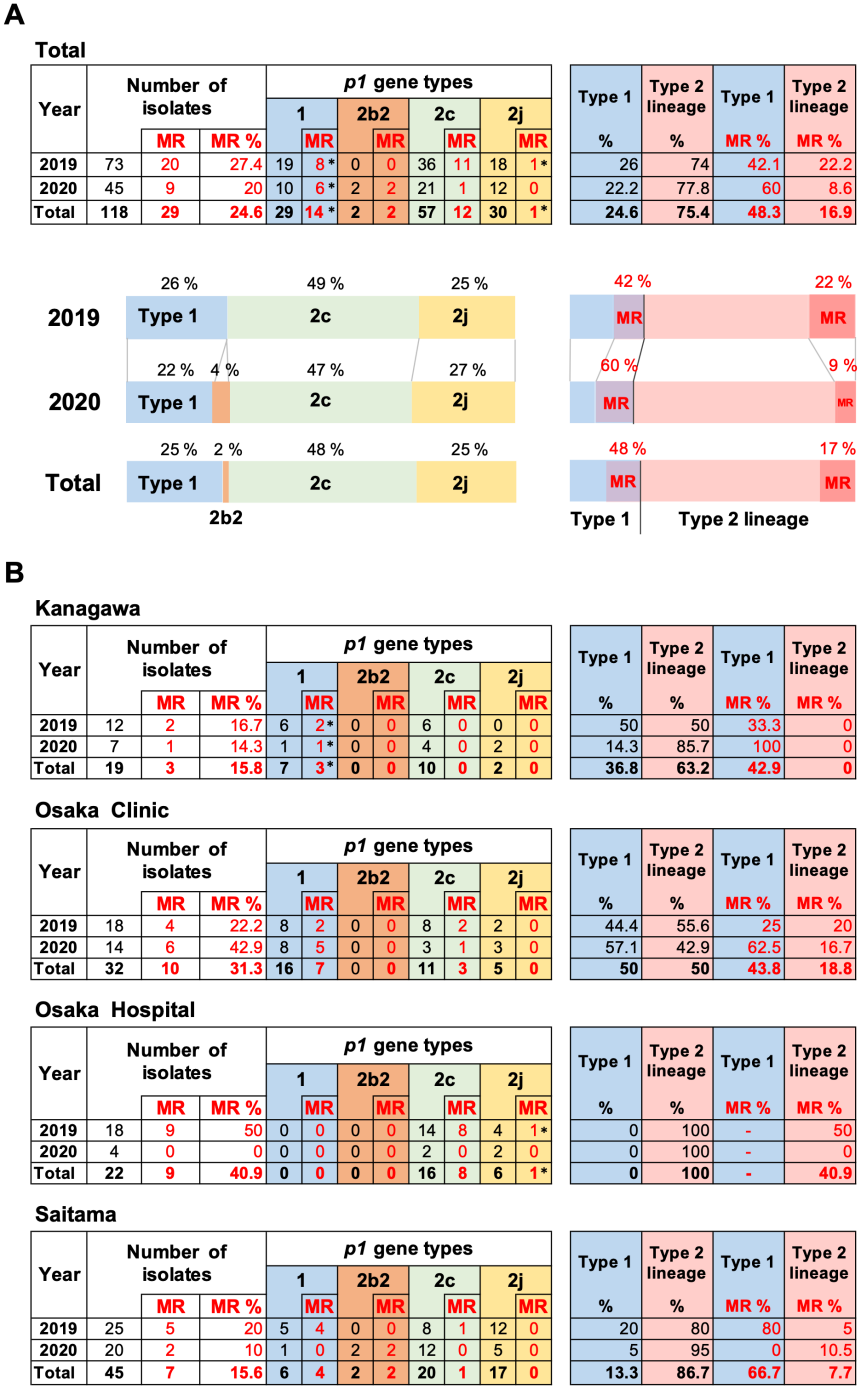
Phylogenetic analysis of type 1 lineage strains. **(A)** Simplified phylogenetic tree of type 1 lineage strains. Four major clades (designated T1-1, T1-2, T1-3, and T1-3R in this study) were identified in the phylogenetic tree of 163 type 1 lineage strains, based on the whole-genome SNPs (Supplementary Figure S1). Representative strains of these 4 clades, M129 (T1-1), Y12-4 (T1-2), KPI-131 (T1-3), and OA-63 (T1-3R) are shown. Y12-4 and OA-63 were genome-sequenced in this study. The MLVA and MLST types of these strains are also shown in green characters in parenthesis. **(B)** Clade-specific SNPs found in the three genes, MPN678 (C435T), MPN607 (G465A), and MPN295 (G135T). These SNPs are recognized by the restriction enzyme *Apo*I. **(C)** Scheme of the phylogenetic analysis. PCR primer sequences for amplification of marker genes (MPN295, MPN607, and MPN678) and locations of SNP sites in the amplicons are shown (the primer sequences used for this PCR are given also in Table 1). **(D)** Discrimination of the phylogenetic clades of the representative type 1 strains (M129, Y12-4, KPI-131, and OA-63) by *Apo*I digestion of the PCR products of MPN678, MPN607, and MPN295 genes. **(E)** 2% agarose-gel electrophoresis patterns of PCR products from the analyzed strains. Electrophoresis patterns before and after *Apo*I digestion are shown. The lane numbers correspond to the F panel. **(F)** List of the 22 type 1 *M. pneumoniae* strains analyzed in this study (also see Supplementary Table S1 and the text).

In this study, the detection rate of type 1 macrolide susceptible (MS) strains was higher (15/29, 51.7%), as compared to that observed in previous studies (Kenri et al., 2020; Ishiguro et al., 2021; Nakamura et al., 2021). Therefore, to explore the phylogenetic relationships between type 1 MR and MS strains, we sequenced the genomes of three type 1 isolates (Y12-4, OA-57, and OA-63) collected in this study, which showed that the MR strain OA-63 belonged to the T1-3R clade of the phylogenetic tree (MLVA type 4572; MLST ST3). In contrast, the MS strains Y12-4 and OA-57 belonged to the T1-2 clade (MLVA type 4573; MLST ST17 and unassigned new ST; Figure 4A; Supplementary Figure S1). These findings suggested that the type 1 MR and MS strains have different phylogenetic backgrounds in this study. To confirm this, we developed a simple phylogenetic analysis method based on the WG-SNP data of 163 type 1 strains (Figures 4B–D; Supplementary Figure S1). We identified three SNPs specific to clades T1-1, T1-2, and T1-3R in the type 1 lineage. Strains belonging to clades T1-1, T1-2, and T1-3R specifically harbored an SNP in the MPN607, MPN678, and MPN295 genes, respectively (Figure 4B). These SNPs were identified using *Apo*I restriction enzyme digestion (Figures 4B–D). Analysis of 22 type 1 strains (isolated in this study at the Osaka and Saitama clinics) showed that 11 MS strains belonged to the T1-2 clade, while 11 MR strains belonged to the T1-3R clade (Figures 4E,F). Empirically, it is known that strains belonging to the T1-2 clade usually have three tandem repeats in the Mpn16 marker of MLVA (Degrange et al., 2009; Supplementary Figure S1); therefore, we also analyzed the MLVA Mpn16 markers of 22 isolates (Supplementary Figure S4). Eleven MS strains harbored three tandem repeats in the Mpn16 region, whereas 11 MR strains had two repeats, further supporting the result that the 11 type 1 MS strains belonged to the T1-2 clade (Supplementary Figure S4). These results suggested that T1-2 clade strains have not acquire MR, as compared to the T1-3R strains in Japan, although a high MR rate of T1-2 clade strains (ST17) was reported in Taiwan (Hung et al., 2021). Probably, T1-2 clade strains had been minor population in Japan and were not exposed to macrolide treatments compared to T1-3R strains in the 2000s. T1-1 or T1-3 clade strains were not found in the 22 type 1 strains in this study (from Osaka and Saitama clinics in 2019 and 2020).

### 3.4. Changes in the amino acid sequences of P1 and P40/P90 proteins caused by type 2c and 2j variations

**Figure 5:**
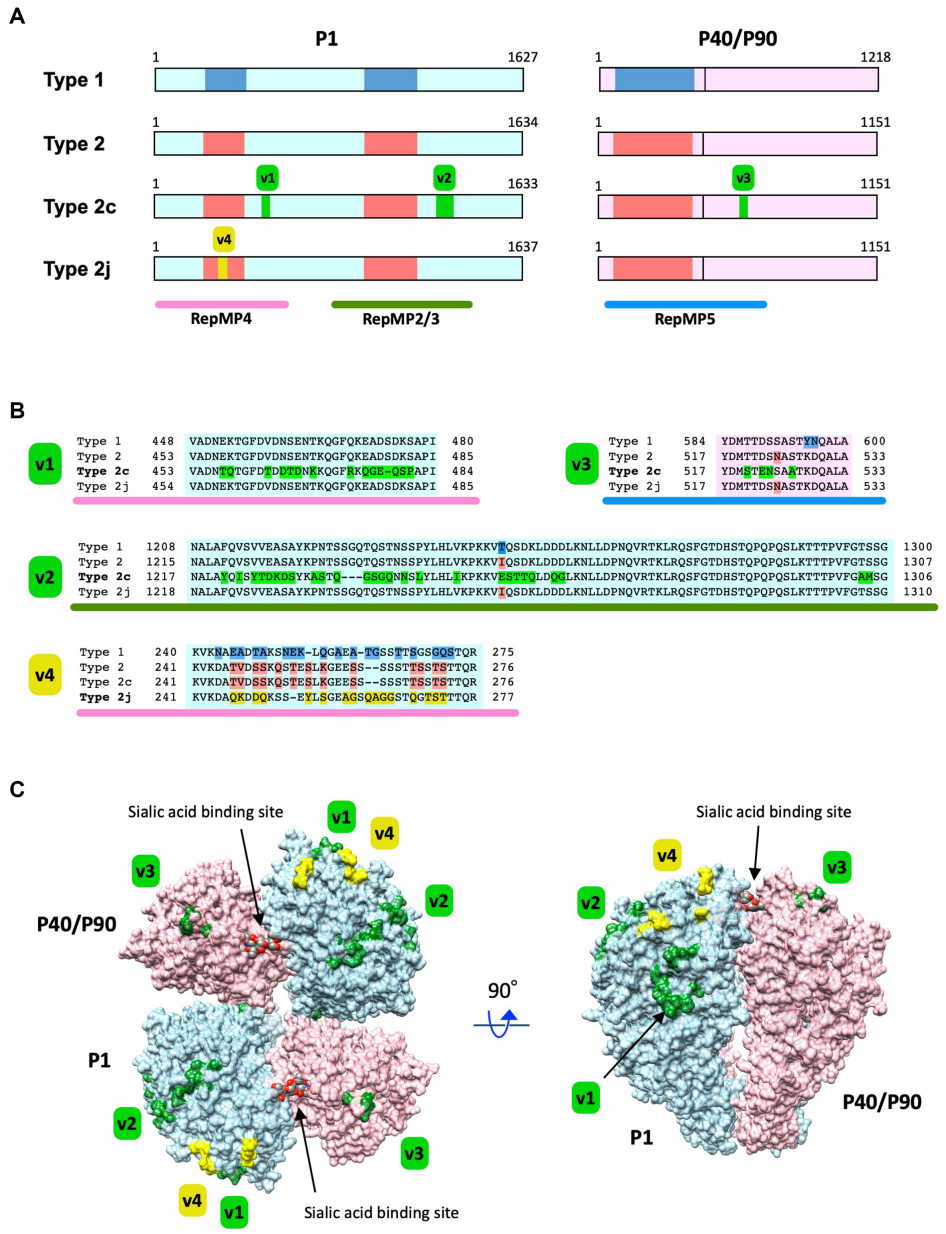
Substitutions of the amino acid residues in P1 and P40/P90 proteins caused by type 2c and 2j variations. **(A)** Locations of the type 2c and 2j variations in the P1 and P40/P90 proteins. The primary structures of the P1 and P40/P90 proteins have been illustrated using light blue and light pink rectangles, respectively. The vertical line in the pink rectangles indicates the cleavage site of P40/P90. The protein type is labeled on the left (Type 1, 2, 2c, and 2j). The blue and red boxes in the rectangles indicate the polymorphic regions between type 1 and 2 lineage sequences. The green boxes (v1–v3) indicate the locations of type 2c variations in the P1 and P40/P90 proteins. The yellow box (v4) indicates the location of the type 2j variation in P1. The RepMP4, RepMP2/3, and RepMP5 repetitive element regions have been indicated using pink, green, and blue bars below, respectively. The illustration has been drawn based on the nucleotide sequence data of type 1, 2, 2c, 2j *p1* and *orf6* genes [GenBank accession nos. M21519 (1054-5937 and 5943-9599), CP010546 (179324-184228 and 184234-187689), AP017319 (179294-184195 and 184201-187656), and LC588412 (1198-6111 and 6117-9572)]. **(B)** Amino acid sequence alignments of the v1, v2, v3, and v4 variation regions. The variant-specific amino acid substitutions have been shown in different colors. **(C)** Three-dimensional structure of the P1 and P40/P90 proteins and variation sites. The P1 and P40/P90 molecules are shown as a hetero-tetrameric adhesin protein complex (nap; Vizarraga et al., 2020). The P1 and P40/P90 molecules are colored light blue and light pink, respectively. The positions of amino acid substitution by the type 2c and 2j variations (v1–v4) are displayed on the nap structure (green and yellow). The image was generated using PDB data 6RC9 and 6RJ1, and the UCSF Chimera software v1.14 (Supplementary Video 1).

This study revealed a high prevalence of type 2c and 2j strains among *M. pneumoniae* isolates in Japan over the recent years. Although the reason for this widespread is unknown, one possible cause is antigenic changes in these variants, particularly in the P1 and P40/P90 proteins. To further investigate this possibility, we inspected the amino acid substitutions in the P1 and P40/P90 proteins in the type 2c and 2j variants. Figures 5A,B show the locations of type 2c and 2j variations in P1 and P40/P90 proteins, as compared with those of classical type 1 and 2. Type 2c has variations in the RepMP4 and RepMP2/3 regions of P1, as compared to those of classical type 2 strains (v1 and v2 in Figure 5). V1 is only present in type 2c, whereas v2 is found in type 2a strains (Zhao et al., 2011; Kenri et al., 2020). Type 2c strains also had a v3 variation in the RepMP5 region of P40/P90 (Figure 5). V3 is in the P90 part of P40/P90 and is present in all type 2c strains analyzed so far (type 2c *orf6* gene; Supplementary Figure S1). V3 was also found in type 2a strains (MLST, ST14 strains); however, some type 2a strains (MLST, ST2, or ST15 strains) did not have v3 (Supplementary Figure S1). Type 2j P1 has variation in the RepMP4 region (v4 in Figure 5). V4 is located in the region that shows sequence variation between type 1 and 2 lineage strains (Figure 5A). The type 2j strains examined had no variation in P40/P90 (Supplementary Figure S1). In v1–v4, some amino acid residues changed their charge and polarity by substitution.

In Figure 5C, the positions of the v1, v2, v3, and v4 variations are displayed in the crystal structures of the P1 and P40/P90 adhesin protein complex (nap). The crystal structures of P1 and P40/P90, which have been elucidated recently, are type 1 (Vizarraga et al., 2020); however, all P1 and P40/P90 subtypes are most likely to have similar protein conformations. Variations v1, v2, v3, and v4 were largely present at the surface of the P1 and P40/P90 molecules. It is possible that these variations can change the antigenicity of the P1 and P40/P90 proteins, which are responsible for the cytadherence of *M. pneumoniae*.

## 4. Discussion

In this study, we showed that type 2 lineage strains were dominant (75.4%, 89/118) among the *M. pneumoniae* isolates collected during the time-period of 2019 and 2020 from patients with respiratory infections in Japan (Figure 1). The most prevalent type 2 lineage was type 2c strain (48% of total isolates, 57/118). Type 2c strains are successful variants that have spread worldwide and are frequently detected in many genotyping studies (Zhao et al., 2011; Edelstein et al., 2016; Gullsby et al., 2019; Kenri et al., 2020; Xiao et al., 2020). Type 2c strains are closely related to type 2a strains, and harbor an additional minor variation in the RepMP4 region of P1 (v1 in Figure 5). Type 2c strains were probably derived from a type 2a strain (MLVA type 3562; MLST ST14; Supplementary Figure S1); however, as compared to that of type 2c, detection of the parent type 2a strain is relatively rare in our epidemiological studies in Japan (Kenri et al., 2020). Another prevalent type 2 lineage strain in this study was type 2j. Type 2j strains may be an intermediate between classical type 2 and type 2 g, in variant development (Figure 1). Type 2j *p1* might have been generated by RepMP4 recombination in a type 2 strain (MLVA type 3662; MLST ST7; Supplementary Figure S1). After this event, additional RepMP2/3 recombination occurred in a type 2j strain, resulting in type 2 g (Kenri et al., 2020). In contrast to the type 2 g strain that was reported in only one isolate in Osaka in 2016 (Katsukawa et al., 2019), type 2j strains have spread widely in Japan and have been clinically prevalent since the middle of the 2010s. To detect type 2j strains, *Mbo*I digestion should be included in the PCR-RFLP genotyping analysis.

The real factors that contribute to type 2c and 2j becoming prevalent strains are unknown; however, several factors may be involved in this phenomenon, including the growth rate of these variants during infections, evasion of herd immunity by antigenic changes, increase in infectivity or colonization ability, or chance. In terms of antigenic changes, the type 2c and 2j strains have amino acid substitutions at the surface of the P1-P40/P90 adhesin complex (nap; Figure 5). Nap is a major antigen of this bacterium and a target of host protective immunity (Dumke et al., 2008; Zhu et al., 2012a,b). Nap surface variations may affect or disturb host immune recognition and provide advantages for evasion of herd immunity. It is also possible that nap surface variations modify sialic acid binding affinity and change the colonization rate of the bacterium at the host surface. Seroepidemiological studies on the variant P1 and P40/P90 proteins are important to discern whether the type 2c and 2j variations are advantageous for survival of *M. pneumoniae* during infection and for becoming prevalent strains. Furthermore, future studies employing modern structural biology techniques may also provide further insights on the effect of nap variations on epitope recognition by host protective immunity and sialic acid receptor binding.

Explanation of the genotype shift mechanism of clinically prevalent strains in terms of the nap antigenic variations has, however, a contradiction in the type 1 lineage, because the type 1 lineage P1 and P40/P90 proteins are less variable, as compared to those of the type 2 lineage (Supplementary Figure S1). The low variation may be partly due to the lower DNA recombination activity in type 1 cells than in type 2 (Krishnakumar et al., 2010). However, the type 1 P40/P90 protein is approximately 70 amino acids longer than that of the type 2 lineage (Ruland et al., 1994). Furthermore, it is also known that this additional 70 amino acid region is structurally disordered and cannot be elucidated by means of structural analysis (Vizarraga et al., 2020). Although the function of this disordered region of the type 1 P40/P90 protein is unknown, there is a need for evaluation of the role of this region in epitope recognition by host immunity in future studies. *Mycoplasma genitalium* P140 and P110 proteins, the homologs of P1 and P40/P90, respectively, do not have disordered regions, as compared to P1 and P40/P90 (Aparicio et al., 2018, 2020). P140 and P110 frequently generate variations, as compared to P1 and P40/P90 (Wood et al., 2020). Thus, disordered amino acid sequence regions might have advantages in host immune evasion without generating sequence variations.

The total MR rate of the 118 strains analyzed in this study was 24.6% (29/118), which is lower than that observed in previous studies and is mainly due to the increased proportion of MS type 2 lineage strains (Figures 2, 3). However, we also found a decreased MR rate of the type 1 strain (14/29, 48.3%) as compared to that observed in previous reports (Katsukawa et al., 2019; Kenri et al., 2020; Ishiguro et al., 2021; Nakamura et al., 2021). The situation of antimicrobial agents consumption under the National Action Plan on Antimicrobial Resistance (since 2016) may also be involved in these decreasing MR trend. Antimicrobial consumption surveillance data^4^ indicates that macrolide sales in Japan was reduced by 21% in 2021 and 39% in 2020, as compared to that in 2013. In addition, tosufloxacin, a fluoroquinolone antimicrobial has been used for treatments of pediatric *M. pneumoniae* pneumonia when macrolides are not effective (Figure 3E). These factors might have contributed to reduce the MR rate of *M. pneumoniae*. However, our present study also showed that type 1 MS strains were phylogenetically different from MR strains (Figure 4; Supplementary Figure S4). In addition, there was a slight increase in the MR rate of the type 2 lineage (15/89, 16.9%; Figures 2A, 3C). These facts cannot be explained only by reduction of clinical macrolides usage. Thus, continuous monitoring is needed to evaluate and understand the correlations between the MR rate of clinical isolates, drug consumption, and genotype shift of circulating strains. The MR rate of the type 2 lineage remains low in Japan; however, a higher MR rate of the type 2 lineage has recently been reported in China and South Korea (Wang et al., 2021; Guo et al., 2022; Lee et al., 2022; Wang et al., 2022). Therefore, it is particularly important to monitor the MR rate of the type 2 lineage strains in Japan.

Since April 2020, when the COVID-19 pandemic started in Japan, there has been a sharp decline in the incidence of *M. pneumoniae* pneumonia (Figure 3E).^5^ This has made it very difficult to obtain *M. pneumoniae* clinical isolates and carry out epidemiological studies. Although it is unknown when and whether the *M. pneumoniae* infections will reappear after the COVID-19 pandemic, there is a possibility of profile changes in the genotype and MR status of *M. pneumoniae* clinical isolates. Thus, it is important to continue extensive efforts in epidemiological studies on *M. pneumoniae*.

## Data availability statement

The datasets presented in this study can be found in online repositories. The names of the repository/repositories and accession number(s) can be found in the article/Supplementary material.

## Author contributions

TK, TY, and KS designed the study. TK, TO, and KS obtained funding. TY, HO, MJ, YO, SA, RS, NI, TO, HF, TH, and HN collected the specimens. TK, HO, MJ, RS, AH, and HF performed the experiments. TK analyzed the data and wrote the draft of the manuscript. All authors contributed to the article and approved the submitted version.

## Funding

This work was supported by grants from Japan Agency for Medical Research and Development (20jk0210004j0101, 18jk0210004j0101, 17jk0210004j0001, 16jk0210004j0001, and 15jk0210004h0027) and was supported in part by a Grants-in-Aid for Scientific Research (15H01337, 20K08171 and 22K07063) from the Ministry of Education, Culture, Sports, Science, and Technology of Japan.

## Conflict of interest

The authors declare that the research was conducted in the absence of any commercial or financial relationships that could be construed as a potential conflict of interest.

## Publisher’s note

All claims expressed in this article are solely those of the authors and do not necessarily represent those of their affiliated organizations, or those of the publisher, the editors and the reviewers. Any product that may be evaluated in this article, or claim that may be made by its manufacturer, is not guaranteed or endorsed by the publisher.

## Footnotes

^1^https://github.com/tseemann/shovill

^2^https://cge.food.dtu.dk/services/CSIPhylogeny/

^3^http://tree.bio.ed.ac.uk/software/figtree

^4^https://amrcrc.ncgm.go.jp/surveillance/Surveillance_en.html

^5^https://www.niid.go.jp/niid/ja/10/2096-weeklygraph/1659-18myco.html
