# Supplementary material for: Genotyping of Mycoplasma pneumoniae strains isolated in Japan during 2019 and 2020: spread of p1 gene type 2c and 2j variant strains
Source: Front Microbiol. 2023 Jun 19;14:1202357. doi: 10.3389/fmicb.2023.1202357 (PMC10316025; doi:10.3389/fmicb.2023.1202357)
Supplement: Supplementary file 1 [file Data_Sheet_1.pdf]

Supplementary Material

Genotyping of *Mycoplasma pneumoniae* strains isolated in Japan during 2019 and 2020: Spread of *p1* gene type 2c and 2j variant strains

Running title: Spread of new *M. pneumoniae* variant

Tsuyoshi Kenri[1*], Tsutomu Yamazaki[2], Hitomi Ohya[3], Michio Jinnai[3], Yoichiro Oda[4], Sadasaburo Asai[5], Rikako Sato[6], Nobuhisa Ishiguro[6], Tomohiro Oishi[7], Atsuko Horino[1], Hiroyuki Fujii[8], Toru Hashimoto[8], Hiroshi Nakajima[9], and Keigo Shibayama[10]

[1]Department of Bacteriology II, National Institute of Infectious Diseases, Tokyo, Japan

[2]Wakaba Children's Clinic, Saitama, Japan

[3]Kanagawa Prefectural Institute of Public Health, Kanagawa, Japan

[4]Chigasaki Municipal Hospital, Kanagawa, Japan

[5]Asai Children's Clinic, Osaka, Japan

[6]Department of Pediatrics, Hokkaido University Graduate School of Medicine, Sapporo, Hokkaido, Japan

[7]Department of Clinical Infectious Diseases, Kawasaki Medical School, Okayama, Japan

[8]Kurashiki Central Hospital, Okayama, Japan

[9]Okayama Prefectural Institute for Environmental Science and Public Health, Okayama, Japan

[10]Department of Bacteriology, Nagoya University Graduate School of Medicine, Nagoya, Japan

***Correspondence:**
Tsuyoshi Kenri
kenri@niid.go.jp

1 Supplementary Figures and Tables

1.1 Supplementary Figures

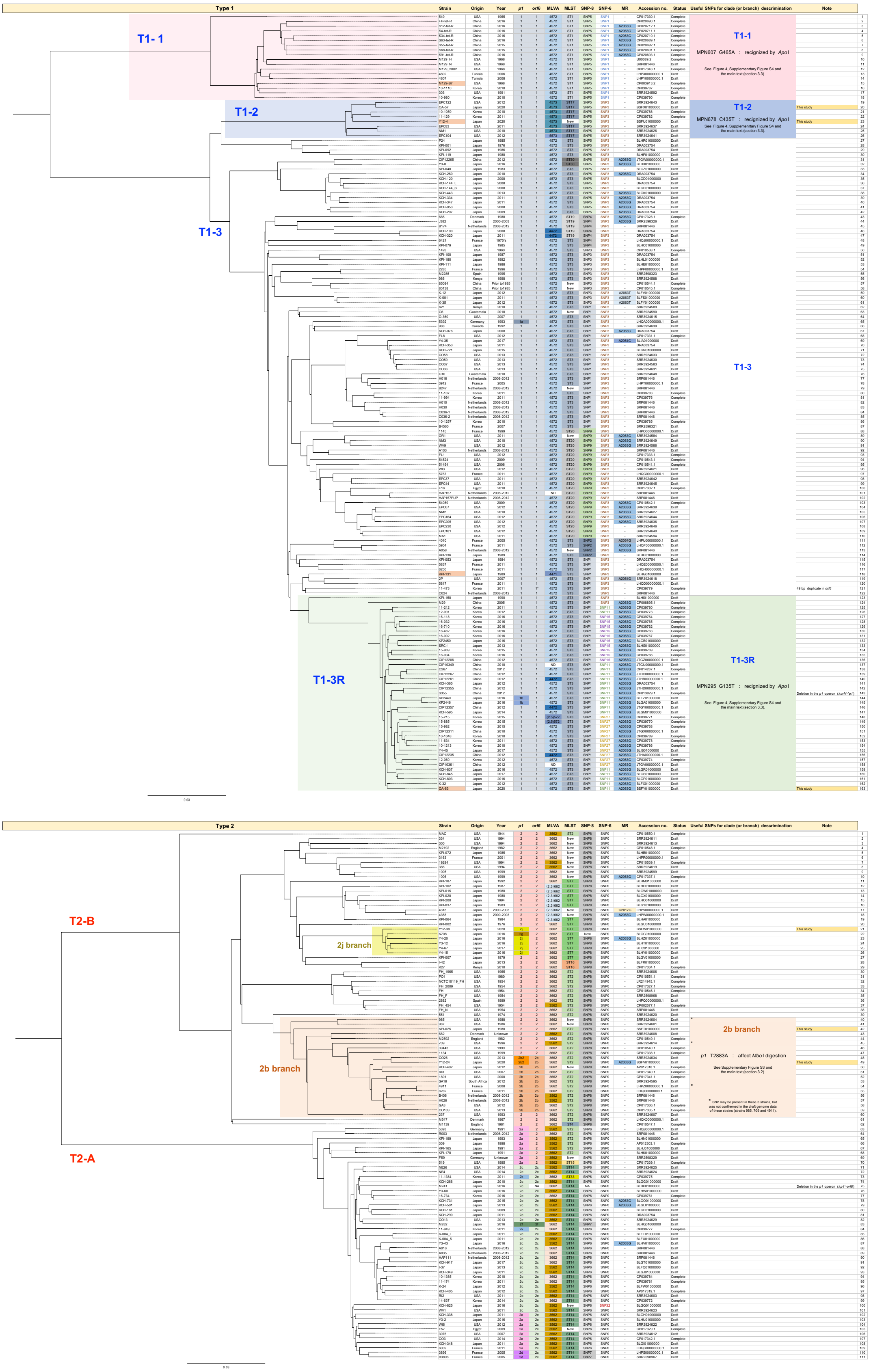

ND : Not determined
NA : Not applicable
NEW : Unassigned new type

Supplementary Figure S1

Phylogenetic tree of *M. pneumoniae* strains based on whole-genome SNP analysis. Phylogenetic tree of (A) 163 type 1 and (B) 111 type 2 lineage strains. The profiles of strains (*p1*, *orf6*, MLVA, MLST, SNP-8, and SNP-6 genotypes), MR, GenBank accession nos., and genome sequencing status are shown on the right of the trees. Information about the useful SNPs for clade (or branch) discrimination is also shown on the right. The MLVA, MLST, SNP-8, and SNP-6 types were determined based on the typing schemes of original reports.

p1 and *orf6* typing : Cousin-Allery, A., Charron, A., de Barbeyrac, B., Fremy, G., Skov Jensen, J., Renaudin, H., and Bebear, C. (2000). Molecular typing of *Mycoplasma pneumoniae* strains by PCR-based methods and pulsed-field gel electrophoresis. Application to French and Danish isolates. *Epidemiol Infect* 124, 103-111.
Kenri, T., Suzuki, M., Sekizuka, T., Ohya, H., Oda, Y., Yamazaki, T., Fujii, H., Hashimoto, T., Nakajima, H., Katsukawa, C., Kuroda, M., and Shibayama, K. (2020). Periodic Genotype Shifts in Clinically Prevalent *Mycoplasma pneumoniae* Strains in Japan. *Front Cell Infect Microbiol* 10, 385. doi: 10.3389/fcimb.2020.00385.
MLVA : Degrange, S., Cazanave, C., Charron, A., Renaudin, H., Bebear, C., and Bebear, C.M. (2009). Development of multiple-locus variable-number tandem-repeat analysis for molecular typing of *Mycoplasma pneumoniae*. *J Clin Microbiol* 47, 914-923. doi: 10.1128/JCM.01935-08.
MLST : Brown, R.J., Holden, M.T., Spiller, O.B., and Chalker, V.J. (2015). Development of a Multilocus Sequence Typing Scheme for Molecular Typing of *Mycoplasma pneumoniae*. *J Clin Microbiol* 53, 3195-3203. doi: 10.1128/JCM.01301-15.
SNP-8 : Touati, A., Blouin, Y., Sirand-Pugnet, P., Renaudin, H., Oishi, T., Vergnaud, G., Bebear, C., and Pereyre, S. (2015). Molecular Epidemiology of *Mycoplasma pneumoniae*: Genotyping Using Single Nucleotide Polymorphisms and SNaPshot Technology. *J Clin Microbiol* 53, 3182-3194. doi: 10.1128/JCM.01156-15.
SNP-6 : Zhao, F., Zhang, J., Wang, X., Liu, L., Gong, J., Zhai, Z., He, L., Meng, F., and Xiao, D. (2021). A multisite SNP genotyping and macrolide susceptibility gene method for *Mycoplasma pneumoniae* based on MALDI-TOF MS. *iScience* 24, 102447. doi: 10.1016/j.isci.2021.102447.

Annual typing number and proportion of *p1* gene types

	Variant	1976	1977	1978	1979	1980	1981	1982	1983	1984	1985	1986	1987	1988	1989	1990	1991	1992	1993	1994	1995	1996	1997	1998	1999	2000	2001	2002	2003	2004	2005	2006	2007	2008	2009	2010	2011	2012	2013	2014	2015	2016	2017	2018	2019	2020	Total	%
T1	1	3	0	0	1	1	0	0	10	7	18	4	12	20	19	7	18	13	4	1	0	0	0	0	0	0	14	26	26	28	22	14	1	20	19	13	115	59	13	15	101	167	26	2	25	10	854	54.1 %
	1b	0	0	0	0	0	0	0	0	0	0	0	0	0	0	0	0	0	0	0	0	0	0	0	0	0	0	0	0	0	0	0	0	0	0	0	0	0	0	0	0	5	0	0	0	0	5	0.3 %
T2	2	1	0	0	3	19	0	0	10	13	2	0	1	0	1	0	0	7	5	12	7	13	8	9	1	24	19	22	7	0	0	0	0	0	0	2	0	0	0	0	0	0	0	0	0	0	186	11.8 %
	2a	0	0	0	0	0	0	0	0	0	0	0	0	0	0	0	2	0	1	0	0	0	0	1	0	0	1	0	5	9	2	0	0	0	0	0	8	0	0	0	0	1	0	0	0	0	30	1.9 %
	2b	0	0	0	0	0	0	0	0	0	0	0	0	0	0	0	0	0	0	0	0	0	0	0	0	0	0	0	0	0	0	0	0	0	0	1	1	1	0	0	0	0	0	0	0	0	3	0.2 %
	2b2	0	0	0	0	0	0	0	0	0	0	0	0	0	0	0	0	0	0	0	0	0	0	0	0	0	0	0	0	0	0	0	0	0	0	0	0	0	0	0	0	0	0	0	0	2	2	0.1 %
	2c	0	0	0	0	0	0	0	0	0	0	0	0	0	0	0	0	0	0	0	0	0	0	0	0	0	0	0	0	0	0	3	0	0	1	4	13	2	6	9	61	102	24	10	39	21	295	18.7 %
	2f	0	0	0	0	0	0	0	0	0	0	0	0	0	0	0	0	0	0	0	0	0	0	0	0	0	0	0	0	0	0	0	0	0	0	0	0	0	0	0	0	1	0	0	0	0	1	0.1 %
	2g	0	0	0	0	0	0	0	0	0	0	0	0	0	0	0	0	0	0	0	0	0	0	0	0	0	0	0	0	0	0	0	0	0	0	0	0	0	0	0	0	1	0	0	0	0	1	0.1 %
	2j	0	0	0	0	0	0	0	0	0	0	0	0	0	0	0	0	0	0	0	0	0	0	0	0	0	0	0	0	0	0	0	0	0	0	0	0	0	0	0	52	68	18	27	24	12	201	12.7 %
Total		4	0	0	4	20	0	0	20	20	20	4	13	20	20	7	20	20	10	13	7	13	8	10	1	24	34	48	38	37	24	17	1	20	20	20	137	62	19	24	214	345	68	39	88	45	**1578**	

Number of MRMP

																																																Total MR	%
T1	1	MR	0	-	-	0	0	-	-	0	0	0	0	0	0	0	0	0	0	0	0	-	-	-	-	-	-	0	0	0	0	0	0	0	13	8	11	97	56	13	15	85	159	24	1	14	6	502	93.7 %
	1b	MR	-	-	-	-	-	-	-	-	-	-	-	-	-	-	-	-	-	-	-	-	-	-	-	-	-	-	-	-	-	-	-	-	-	-	-	-	-	-	-	-	5	-	-	-	-	5	0.9 %
T2	2	MR	0	-	-	0	0	-	-	0	0	0	-	0	-	0	-	-	0	0	0	0	0	0	0	0	0	0	0	0	-	-	-	-	-	-	0	-	-	-	-	-	-	-	-	-	-	0	0 %
	2a	MR	-	-	-	-	-	-	-	-	-	-	-	-	-	-	-	0	-	0	-	-	-	-	0	-	-	0	-	0	0	0	-	-	-	-	-	0	-	-	-	-	0	-	-	-	-	0	0 %
	2b	MR	-	-	-	-	-	-	-	-	-	-	-	-	-	-	-	-	-	-	-	-	-	-	-	-	-	-	-	-	-	-	-	-	-	-	0	0	0	-	-	-	-	-	-	-	-	0	0 %
	2b2	MR	-	-	-	-	-	-	-	-	-	-	-	-	-	-	-	-	-	-	-	-	-	-	-	-	-	-	-	-	-	-	-	-	-	-	-	-	-	-	-	-	-	-	-	-	2	2	0.4 %
	2c	MR	-	-	-	-	-	-	-	-	-	-	-	-	-	-	-	-	-	-	-	-	-	-	-	-	-	-	-	-	-	-	0	-	-	0	0	1	0	0	0	8	1	2	0	11	1	24	4.5 %
	2f	MR	-	-	-	-	-	-	-	-	-	-	-	-	-	-	-	-	-	-	-	-	-	-	-	-	-	-	-	-	-	-	-	-	-	-	-	-	-	-	-	-	0	-	-	-	-	0	0 %
	2g	MR	-	-	-	-	-	-	-	-	-	-	-	-	-	-	-	-	-	-	-	-	-	-	-	-	-	-	-	-	-	-	-	-	-	-	-	-	-	-	-	-	0	-	-	-	-	0	0.0 %
	2j	MR	-	-	-	-	-	-	-	-	-	-	-	-	-	-	-	-	-	-	-	-	-	-	-	-	-	-	-	-	-	-	-	-	-	-	-	-	-	-	-	0	2	0	0	1	0	3	0.6 %
MR Total			0	-	-	0	0	-	-	0	0	0	0	0	0	0	0	0	0	0	0	0	0	0	0	0	0	0	0	0	0	0	0	0	13	8	11	98	56	13	15	93	167	26	1	26	9	**536**	

Rate of MRMP (%)

																																																Total MR %
T1	1	MR %	0	-	-	0	0	-	-	0	0	0	0	0	0	0	0	0	0	0	0	-	-	-	-	-	-	0	0	0	0	0	0	0	65	42.1	84.6	84.3	94.9	100	100	84.2	95.2	92.3	50	56	60	58.8 %
	1b	MR %	-	-	-	-	-	-	-	-	-	-	-	-	-	-	-	-	-	-	-	-	-	-	-	-	-	-	-	-	-	-	-	-	-	-	-	-	-	-	-	-	100	-	-	-	-	100 %
T2	2	MR %	0	-	-	0	0	-	-	0	0	0	-	0	-	0	-	-	0	0	0	0	0	0	0	0	0	0	0	0	-	-	-	-	-	-	0	-	-	-	-	-	-	-	-	-	-	0 %
	2a	MR %	-	-	-	-	-	-	-	-	-	-	-	-	-	-	-	0	-	0	-	-	-	-	0	-	-	0	-	0	0	0	-	-	-	-	-	0	-	-	-	-	0	-	-	-	-	0 %
	2b	MR %	-	-	-	-	-	-	-	-	-	-	-	-	-	-	-	-	-	-	-	-	-	-	-	-	-	-	-	-	-	-	-	-	-	-	0	0	0	-	-	-	-	-	-	-	-	0 %
	2b2	MR %	-	-	-	-	-	-	-	-	-	-	-	-	-	-	-	-	-	-	-	-	-	-	-	-	-	-	-	-	-	-	-	-	-	-	-	-	-	-	-	-	-	-	-	-	100	100 %
	2c	MR %	-	-	-	-	-	-	-	-	-	-	-	-	-	-	-	-	-	-	-	-	-	-	-	-	-	-	-	-	-	-	0	-	-	0	0	0	0	0	0	13.1	1.0	8.3	0	28.2	4.8	8.1 %
	2f	MR %	-	-	-	-	-	-	-	-	-	-	-	-	-	-	-	-	-	-	-	-	-	-	-	-	-	-	-	-	-	-	-	-	-	-	-	-	-	-	-	-	0	-	-	-	-	0 %
	2g	MR %	-	-	-	-	-	-	-	-	-	-	-	-	-	-	-	-	-	-	-	-	-	-	-	-	-	-	-	-	-	-	-	-	-	-	-	-	-	-	-	-	0	-	-	-	-	0 %
	2j	MR %	-	-	-	-	-	-	-	-	-	-	-	-	-	-	-	-	-	-	-	-	-	-	-	-	-	-	-	-	-	-	-	-	-	-	-	-	-	-	-	0	2.9	0	0	4.2	0	1.5 %
Total		MR %	0	-	-	0	0	-	-	0	0	0	0	0	0	0	0	0	0	0	0	0	0	0	0	0	0	0	0	0	0	0	0	0	65	40	55	71.5	90.3	68.4	62.5	43.5	48.4	38.2	2.6	29.5	20	34 %

Ratio of Type 1 and 2 (T1 and T2) lineage and MRMP (number and rate)

																																															Total (number and %)
T1		3	0	0	1	1	0	0	10	7	18	4	12	20	19	7	18	13	4	1	0	0	0	0	0	0	14	26	26	28	22	14	1	20	19	13	115	59	13	15	101	172	26	2	25	10	859
T2		1	0	0	3	19	0	0	10	13	2	0	1	0	1	0	2	7	6	12	7	13	8	10	1	24	20	22	12	9	2	3	0	0	1	7	22	3	6	9	113	173	42	37	63	35	719
Total		4	0	0	4	20	0	0	20	20	20	4	13	20	20	7	20	20	10	13	7	13	8	10	1	24	34	48	38	37	24	17	1	20	20	20	137	62	19	24	214	345	68	39	88	45	1578
T1	%	75	-	-	25	5	-	-	50	35	90	100	92	100	95	100	90	65	40	8	0	0	0	0	0	0	41	54	68	76	92	82	100	100	95	65	84	95	68	63	47	50	38	5	28	22	54 %
T2	%	25	-	-	75	95	-	-	50	65	10	0	8	0	5	0	10	35	60	92	100	100	100	100	100	100	59	46	32	24	8	18	0	0	5	35	16	5	32	38	53	50	62	95	72	78	46 %

																																															Total MR (number and %)
T1	MR	0	-	-	0	0	-	-	0	0	0	0	0	0	0	0	0	0	0	0	-	-	-	-	-	-	0	0	0	0	0	0	0	13	8	11	97	56	13	15	85	164	24	1	14	6	507
T2	MR	0	-	-	0	0	-	-	0	0	0	-	0	-	0	-	0	0	0	0	0	0	0	0	0	0	0	0	0	0	0	0	-	-	0	0	1	0	0	0	8	3	2	0	12	3	29
Total	MR	0	-	-	0	0	-	-	0	0	0	0	0	0	0	0	0	0	0	0	0	0	0	0	0	0	0	0	0	0	0	0	0	13	8	11	98	56	13	15	93	167	26	1	26	9	536
T1	MR %	0	-	-	0	0	-	-	0	0	0	0	0	0	0	0	0	0	0	0	-	-	-	-	-	-	0	0	0	0	0	0	0	65	42	85	84	95	100	100	84	95	92	50	56	60	59 %
T2	MR %	0	-	-	0	0	-	-	0	0	0	-	0	-	0	-	0	0	0	0	0	0	0	0	0	0	0	0	0	0	0	0	-	0	0	0	4.5	0	0	0	7.1	1.7	4.8	0	19	8.6	4 %
Total	MR %	0	-	-	0	0	-	-	0	0	0	0	0	0	0	0	0	0	0	0	0	0	0	0	0	0	0	0	0	0	0	0	0	65	40	55	72	90	68	63	43	48	38	2.6	30	20	34 %

Data source

1976-1994 Sasaki et al., 1996 (1)
1995-2005 Kenri et al., 2008 (2)
2006-2019 Kenri et al., 2020 (5)
2011-2017 Katsukawa et al., 2019 (4)
2019-2020 This study
2011 Horino et al., 2012 (3)

Total

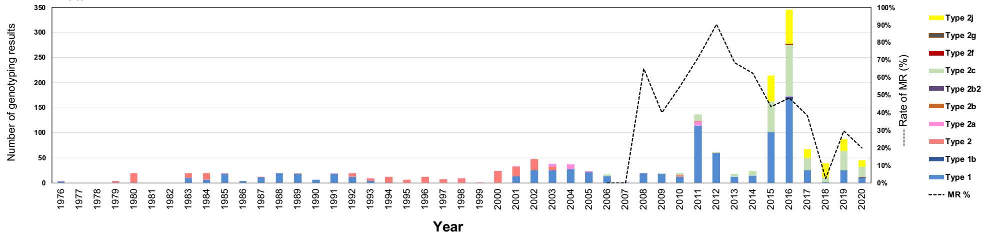

Type 1 lineage

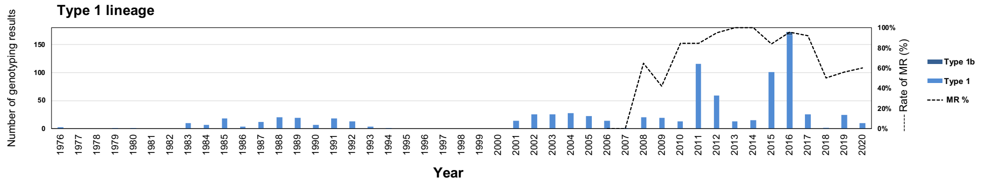

Type 2 lineage

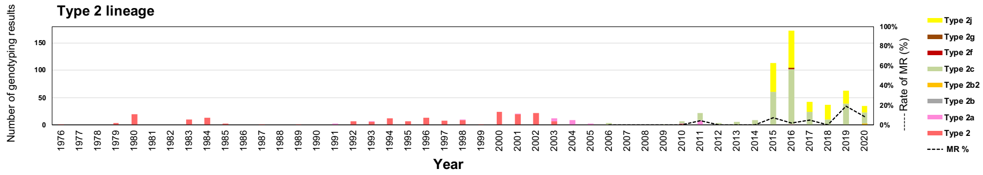

Ratio

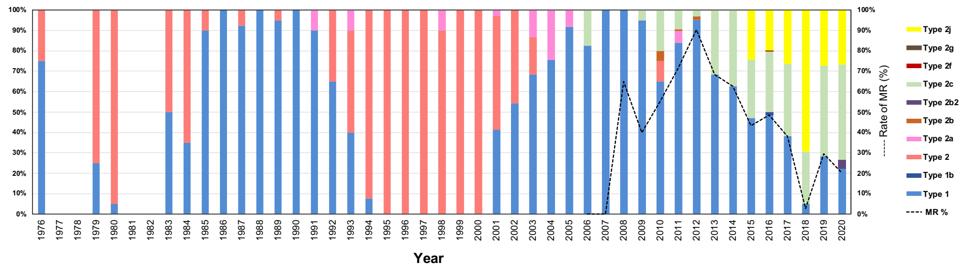

Supplementary Figure S2

Time trend of the *p1* genotype of *M. pneumoniae* clinical isolates in Japan. Genotyping data of our previous reports, Sasaki et al., 1996 (1), Kenri et al., 2008 (2), Horino et al., 2012 (3), Katsukawa et al., 2019 (4), and Kenri et al., 2020 (5) were revised and integrated with the data of this study.

1: Sasaki T, Kenri T, Okazaki N, Iseki M, Yamashita R, Shintani M, Sasaki Y, Yayoshi M. (1996). Epidemiological study of *Mycoplasma pneumoniae* infections in Japan based on PCR-restriction fragment length polymorphism of the P1 cytadhesin gene. J Clin Microbiol. 1996, 34, 447-9.
2: Kenri T, Okazaki N, Yamazaki T, Narita M, Izumikawa K, Matsuoka M, Suzuki S, Horino A, Sasaki T. (2008). Genotyping analysis of *Mycoplasma pneumoniae* clinical strains in Japan between 1995 and 2005: Type shift phenomenon of *M. pneumoniae* clinical strains. J Med Microbiol. 57, 469-75.
3: Horino A, Kenri T, Sasaki Y, Suzuki S, Shibayama K, Yasui Y, and Taniguchi K (2012). Analysis of clinical isolates from hospitalized patients of *Mycoplasma pneumoniae* infection during the 2011 epidemic. Infectious agents surveillance report (IASR), 33(10), 264-265. https://www.niid.go.jp/niid/ja/typhi-m/iasr-reference/2123-related-articles/related-articles-392/2709-dj3922.html
4: Katsukawa, C., Kenri, T., Shibayama, K., and Takahashi, K. (2019).Genetic characterization of *Mycoplasma pneumoniae* isolated in Osaka between 2011 and 2017: Decreased detection rate of macrolide-resistance and increase of *p1* gene type 2 lineage strains. PLoS One 14, e0209938.
5: Kenri T, Suzuki M, Sekizuka T, Ohya H, Oda Y, Yamazaki T, Fujii H, Hashimoto T, Nakajima H, Katsukawa C, Kuroda M, and Shibayama K (2020). Periodic Genotype Shifts in Clinically Prevalent *Mycoplasma pneumoniae* Strains in Japan. Front Cell Infect Microbiol 10, 385. doi: 10.3389/fcimb.2020.00385.

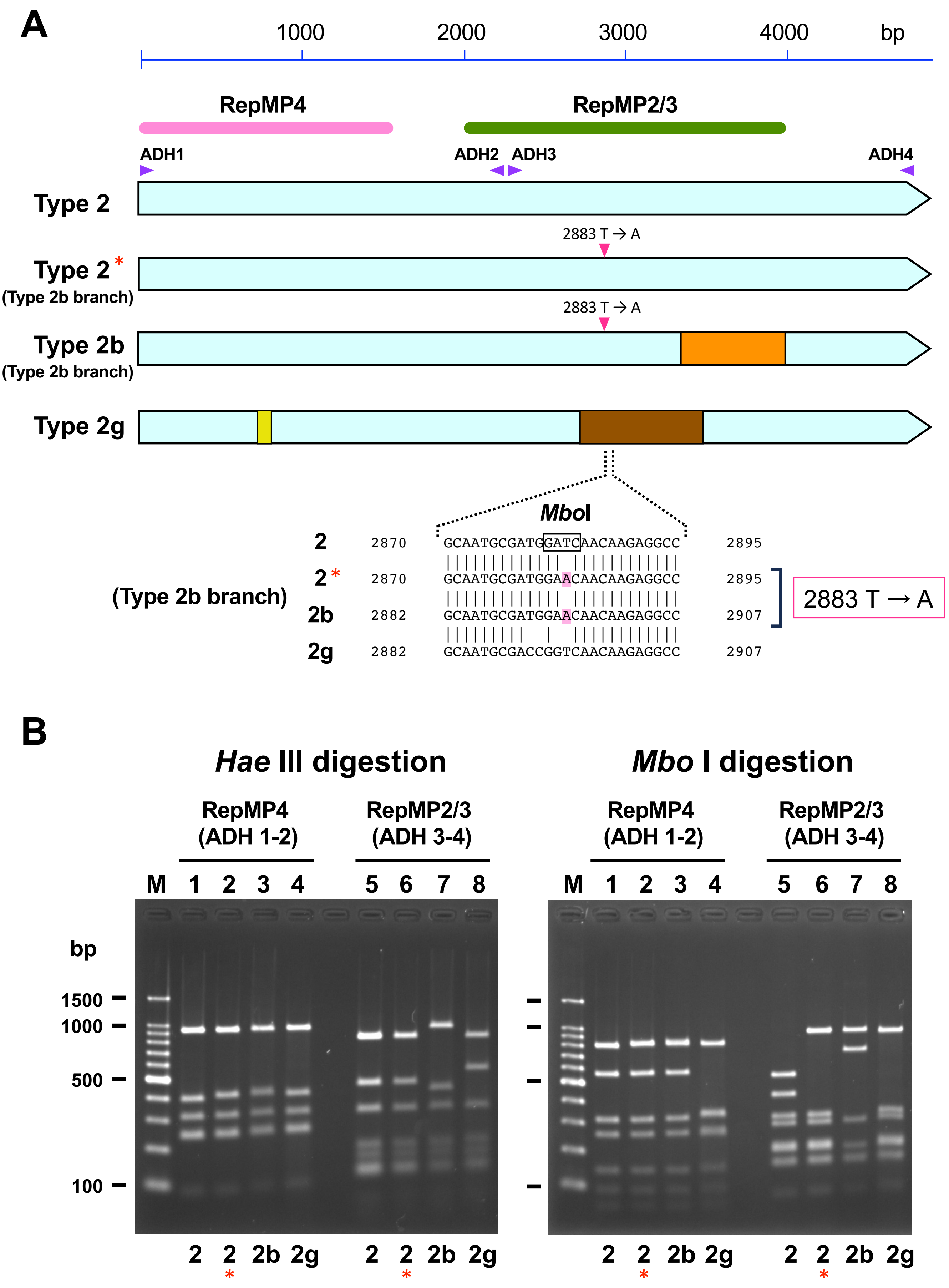

Supplementary Figure S3

P1 gene typing of type 2, 2 (2b branch), 2b, and 2g strains using PCR-RFLP analysis. **(A)** Schematic illustration of *p1* gene variants. Light blue arrows show type 2, 2 (2b branch), 2b, and 2g *p1* genes. The orange, yellow, and brown boxes in the arrows indicate the location of variation sites of type 2b and 2g *p1*. Presence of the SNP (2883 T to A) has been indicated using pink triangles. The sequences of this SNP region are shown below. The pink and green bars indicate the locations of RepMP4 and RepMP2/3 regions, respectively. Small purple triangles indicate the positions of the ADH1, ADH2, ADH3, and ADH4 primers for PCR-RFLP analysis. **(B)** Band patterns of PCR-RFLP analysis of 2, 2 (2b branch), 2b, and 2g strains, as obtained upon 2% agarose-gel electrophoresis. The 2 (2b branch) strain was marked using a red asterisk. The left and the right panels show the *Hae*III and *Mbo*I digestion patterns of the same samples, respectively. The RepMP4 region (ADH1-2 amplicon) of the FH (type 2), KPI-025 (type 2 in 2b branch), KCH-405 (type 2b), and K708 (type 2g) strains has been analyzed in lanes 1, 2, 3, and 4, respectively (see also Supplementary Figure S1). The RepMP2/3 region (ADH3-4 amplicon) of the same strains has been analyzed in lanes 5, 6, 7, and 8, respectively. Lane M shows the 100 bp ladder DNA size marker.

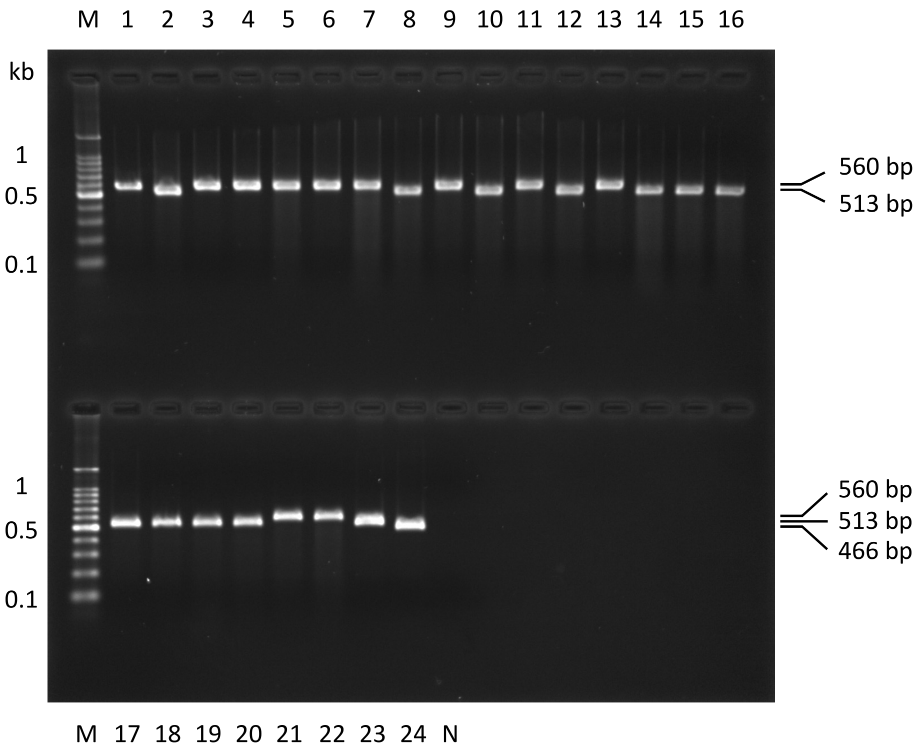

Lane No.	Isolate Name	*p1* type	Origin	Year	MR mutations	MLVA	**Mpn16 Marker repeat number**
1	OA-1	1	Osaka	2019	-		**3**
2	OA-7	1	Osaka	2019	A2063G		**2**
3	OA-15	1	Osaka	2019	-		**3**
4	OA-21	1	Osaka	2019	-		**3**
5	OA-22	1	Osaka	2019	-		**3**
6	OA-25	1	Osaka	2019	-		**3**
7	OA-28	1	Osaka	2019	-		**3**
8	OA-33	1	Osaka	2019	A2063G		**2**
9	OA-38	1	Osaka	2020	-		**3**
10	OA-43	1	Osaka	2020	A2063G		**2**
11	OA-47	1	Osaka	2020	-		**3**
12	OA-49	1	Osaka	2020	A2063G		**2**
13	OA-57	1	Osaka	2020	-	4573	**3**
14	OA-62	1	Osaka	2020	A2063G		**2**
15	OA-63	1	Osaka	2020	A2063G	4572	**2**
16	OA-65	1	Osaka	2020	A2063G		**2**
17	Y11-3	1	Saitama	2019	A2063G		**2**
18	Y11-4	1	Saitama	2019	A2063G		**2**
19	Y11-8	1	Saitama	2019	A2063G		**2**
20	Y11-12	1	Saitama	2019	A2063G		**2**
21	Y11-52	1	Saitama	2019	-		**3**
22	Y12-4	1	Saitama	2020	-	4573	**3**
23	M129	1	USA	1968	-	4572	**2**
24	KPI-131	1	Kanagawa	1989	-	4471	**1**
M	Size Marker (100 bp DNA ladder)						
N	Negative control (blank)						

Row 13 (OA-57): ← Genome sequenced in this study
Accession no. BSFX01000000

Row 15 (OA-63): ← Genome sequenced in this study
Accession no. BSFY01000000

Row 22 (Y12-4): ← Genome sequenced in this study
Accession no. BSFU01000000

PCR primer set used for amplification

Mpn16-FS TGATCCCAACAGTAAACCCT
Mpn16-RS TTCAAAGTAAGCAGATGTAC

PCR product size

Repeat number = 3 560 bp
Repeat number = 2 513 bp
Repeat number = 1 466 bp

Supplementary Figure S4

Analysis of the Mpn16 marker of MLVA in 22 type 1 *M. pneumoniae* strains collected in this study (also see Supplementary Table S1 and the main text). For comparison, two well-characterized type 1 strains M129 and KPI-131 were also analyzed (see Supplementary Figure S1). The PCR primer sequences used in this analysis (Mpn16-FS and Mpn16-FR) and the theoretical size of PCR products are shown. 0.8% agarose-gel electrophoresis was used for the analysis.

1.2 Supplementary Tables

Supplementary Table S1. *M. pneumoniae* isolates collected and analyzed in this study.

Kanagawa (19 isolates)

No.	Isolate Name	*p1* type	MR mutations	Date of sample collection yyyy/mm/dd	Patient Age	Patient Sex	Diagnosis	Specimen	Hospitalization
1	KP2521	1	-	2019/8/14	7y	F	Pneumonia	TS	No
2	KP2522	2c	-	2019/10/9	15y	M	Pneumonia	TS	No
3	KP2523	2c	-	2019/11/18	9y	F	Pneumonia	TS	No
4	KP2524	2c	-	2019/11/27	4y	F	Pneumonia	TS	No
5	KP2525	2c	-	2019/12/2	7y	M	Pneumonia	TS	No
6	KP2527	1	A2063G	2019/12/16	32y	F	Pneumonia	TS	No
7	KP2528	1	-	2019/12/17	8y	F	Pneumonia	TS	No
8	KP3280	2c	-	2019/8/26	12y	M	Pneumonia	TS	No
9	KP3281	1	-	2019/11/14	7y	M	Pneumonia	TS	No
10	KP3282	2c	-	2019/12/5	6y	M	Pneumonia	TS	No
11	KP3283	1	A2064G	2019/12/6	6y	F	Pneumonia	TS	No
12	KP3284	1	-	2019/12/11	1y	M	Pneumonia	TS	No
13	KP2530	2c	-	2020/1/14	8y	M	Pneumonia	TS	No
14	KP2531	2j	-	2020/2/3	5y	M	Pneumonia	TS	No
15	KP2532	2c	-	2020/2/8	17y	F	Pneumonia	TS	No
16	KP2533	2c	-	2020/2/18	10y	M	Pneumonia	TS	No
17	KP2534	2j	-	2020/2/27	9y	M	Pneumonia	TS	No
18	KP3286	1	A2064G	2020/1/31	5y	F	Bronchitis	TS	No
19	KP3287	2c	-	2020/2/3	11y	F	Pneumonia	TS	No

Number of swab specimen : 23
Culture positive rate : 83 % (19/23)

Specimen
TS : Throat swab

Osaka Clinic (32 isolates)

No.	Isolate Name	*p1* type	MR mutations	Mpn16 (MLVA)	Clade	Date of sample collection yyyy/mm/dd	Patient Age	Patient Sex	Diagnosis	Specimen	Hospitalization	Onset of disease yyyy/mm/dd	NAT (LAMP) test
1	OA-1	1	-	3	T1-2	2019/10/29	11 y	F	Pneumonia	TS	No	2019/10/25	+
2	OA-3	2j	-		T2-B	2019/8/8	5 y	F	Bronchitis	TS	No	2019/8/5	+
3	OA-7	1	A2063G	2	T1-3R	2019/7/29	2 y	M	Bronchitis	TS	No	2019/7/29	+
4	OA-15	1	-	3	T1-2	2019/10/21	12 y	F	Bronchitis	TS	No	2019/10/16	+
5	OA-16	2c	-		T2-A	2019/10/18	7 y	F	Bronchitis	TS	No	2019/10/16	+
6	OA-18	2c	-		T2-A	2019/10/21	7 y	M	Pneumonia	TS	No	2019/10/15	+
7	OA-19	2c	-		T2-A	2019/11/12	2 y	M	Bronchitis	TS	No	2019/11/9	+
8	OA-21	1	-	3	T1-2	2019/11/11	12 y	M	Bronchitis	TS	No	2019/11/8	+
9	OA-22	1	-	3	T1-2	2019/11/12	7 y	M	Bronchitis	TS	No	2019/11/11	+
10	OA-23	2c	-		T2-A	2019/11/14	10 y	F	Pneumonia	TS	No	2019/11/12	+
11	OA-25	1	-	3	T1-2	2019/11/25	9 y	F	Bronchitis	TS	No	2019/11/20	-
12	OA-26	2c	A2063G		T2-A	2019/12/2	5 y	F	Bronchitis	TS	No	2019/11/30	+
13	OA-28	1	-	3	T1-2	2019/12/3	8 y	M	Bronchitis	TS	No	2019/11/27	+
14	OA-29	2j	-		T2-B	2019/12/10	12 y	M	Bronchitis	TS	No	2019/12/6	+
15	OA-32	2c	-		T2-A	2019/12/17	7 y	M	Bronchitis	TS	No	2019/12/11	+
16	OA-33	1	A2063G	2	T1-3R	2019/12/17	7 y	F	Bronchitis	TS	No	2019/12/15	+
17	OA-34	2c	-		T2-A	2019/12/19	7 y	F	Bronchitis	TS	No	2019/12/12	+
18	OA-37	2c	A2063G		T2-A	2019/12/27	8 y	F	Bronchitis	TS	No	2019/12/26	+
19	OA-38	1	-	3	T1-2	2020/1/6	12 y	M	Bronchitis	TS	No	2020/1/5	+
20	OA-39	2j	-		T2-B	2020/1/7	9 y	M	Bronchitis	TS	No	2020/1/6	+
21	OA-41	2j	-		T2-B	2020/1/12	10 y	M	Bronchitis	TS	No	2020/1/6	+
22	OA-42	2c	A2063G		T2-A	2020/1/14	6 y	M	Bronchitis	TS	No	2020/1/11	+
23	OA-43	1	A2063G	2	T1-3R	2020/1/18	7 y	M	Bronchitis	TS	No	2020/1/12	+
24	OA-47	1	-	3	T1-2	2020/2/3	7 y	F	Bronchitis	TS	No	2020/1/31	+
25	OA-49	1	A2063G	2	T1-3R	2020/2/9	10 y	F	Pneumonia	TS	No	2020/2/1	+
26	OA-54	2c	-		T2-A	2020/3/2	12 y	F	Bronchitis	TS	No	2020/2/29	+
27	OA-55	2j	-		T2-B	2020/3/5	7 y	M	Bronchitis	TS	No	2020/3/4	+
28	OA-57	1	-	3	T1-2	2020/3/9	7 y	F	Bronchitis	TS	No	2020/3/7	+
29	OA-58	2c	-		T2-A	2020/3/12	3 y	F	Bronchitis	TS	No	2020/3/7	+
30	OA-62	1	A2063G	2	T1-3R	2020/3/27	5 y	M	Bronchitis	TS	No	2020/3/24	+
31	OA-63	1	A2063G	2	T1-3R	2020/4/7	6 y	M	Bronchitis	TS	No	2020/4/1	+
32	OA-65	1	A2063G	2	T1-3R	2020/4/17	3 y	F	Bronchitis	TS	No	2020/4/16	+

Number of swab specimen : 60
Culture positive rate : 53 % (32/60)

Specimen
TS : Throat swab

Osaka Hospital (22 isolates)

No.	Isolate Name	*p1* type	MR mutations	Date of sample collection yyyy/mm/dd	Patient Age	Patient Sex	Diagnosis	Specimen	Hospitalization
1	KT-6	2c	A2063G	2019/8/1	5. 2 y	F	Pneumonia	SS	Yes (9 days)
2	KT-7	2c	—	2019/8/5	12. 8 y	F	Pneumonia	SS	No
3	KT-10	2c	A2063G	2019/8/27	8. 7 y	F	Pneumonia	SS	No
4	KT-13	2c	A2063G	2019/9/17	5 y	M	Pneumonia	SS	No
5	KT-14	2c	—	2019/9/19	0 y	F	Bronchitis	SS	No
6	KT-18	2j	—	2019/10/7	4 y	F	Pneumonia	SS	No
7	KT-19	2j	A2063T	2019/10/7	2 y	M	Pneumonia	SS	Yes (6 days)
8	KT-20	2c	A2063G	2019/10/9	1 y	F	Pneumonia	SS	Yes (6 days)
9	KT-22	2c	—	2019/10/11	14 y	F	Pneumonia	SS	Yes (5 days)
10	KT-28	2c	A2063G	2019/10/18	9 y	M	Pneumonia	SS	No
11	KT-29	2c	A2063G	2019/10/18	5 y	M	Bronchitis	SS	No
12	KT-30	2c		2019/10/19	12 y	M	Pneumonia	SS	Yes (3 days)
13	KT-31	2c	A2063G	2019/10/21	4 y	F	Pneumonia	SS	No
14	KT-35	2j	—	2019/10/28	12 y	F	Pneumonia	SS	No
15	KT-39	2c	A2063G	2019/11/11	7 y	M	Pneumonia	SS	No
16	KT-43	2c	—	2019/11/16	17 y	F	Pneumonia	SS	No
17	KT-46	2j	—	2019/11/22	5 y	M	Pneumonia	SS	No
18	KT-49	2c	—	2019/11/30	22 y	M	Pneumonia	S	No
19	KT-54	2j	—	2020/2/1	6 y	F	Pneumonia	SS	No
20	KT-58	2c	—	2020/3/2	3 y	M	Pneumonia	SS	No
21	KT-59	2j	—	2020/3/6	7 y	M	Pneumonia	SS	No
22	KT-60	2c	—	2020/3/21	8 y	M	Pneumonia	SS	No

Number of specimen : 60
Culture positive rate : 37 % (22/60)

Specimen
SS : Suction sputum
S : Sputum

Saitama (45 isolates)

No.	Isolate Name	*p1* type	MR mutations	Mpn16 (MLVA)	Clade	Date of sample collection yyyy/mm/dd	Patient Age	Patient Sex	Diagnosis	Specimen	Hospitalization
1	Y11-1	2c	-		T2-A	2019/8/9	6 y	F	Pneumonia	TS	No
2	Y11-3	1	A2063G	2	T1-3R	2019/8/13	41 y	M	Bronchitis	TS	No
3	Y11-4	1	A2063G	2	T1-3R	2019/8/15	10 y	M	Bronchitis	TS	No
4	Y11-5	2j	-		T2-B	2019/8/15	8 y	F	Bronchitis	TS	No
5	Y11-8	1	A2063G	2	T1-3R	2019/8/26	10 y	M	Pneumonia	TS	No
6	Y11-12	1	A2063G	2	T1-3R	2019/8/30	44 y	M	Bronchitis	TS	No
7	Y11-16	2j	-		T2-B	2019/9/9	8 y	M	Bronchitis	TS	No
8	Y11-18	2j	-		T2-B	2019/9/9	44 y	M	Pneumonia	TS	No
9	Y11-26	2c	-		T2-A	2019/9/24	8 y	M	Pneumonia	TS	No
10	Y11-29	2c	-		T2-A	2019/9/26	8 y	M	Pneumonia	TS	No
11	Y11-30	2j	-		T2-B	2019/9/30	4 y	F	Pneumonia	TS	No
12	Y11-33	2j	-		T2-B	2019/10/11	6 y	M	Pneumonia	TS	No
13	Y11-36	2j	-		T2-B	2019/10/17	37 y	M	Bronchitis	TS	No
14	Y11-37	2j	-		T2-B	2019/10/21	7 y	F	Bronchitis	TS	No
15	Y11-39	2j	-		T2-B	2019/10/26	3 y	M	Pneumonia	TS	No
16	Y11-42	2j	-		T2-B	2019/11/7	10 y	M	Pneumonia	TS	No
17	Y11-43	2c	A2063G		T2-A	2019/11/8	2 y	M	Pneumonia	TS	No
18	Y11-44	2c	-		T2-A	2019/11/8	7 y	M	Bronchitis	TS	No
19	Y11-49	2j	-		T2-B	2019/11/26	6 y	M	Pneumonia	TS	No
20	Y11-52	1	-	3	T1-2	2019/12/9	8 y	F	Pneumonia	TS	No
21	Y11-55	2c	-		T2-A	2019/12/14	10 y	M	Bronchitis	TS	No
22	Y11-59	2j	-		T2-B	2019/12/17	10 y	M	Bronchitis	TS	No
23	Y11-61	2c	-		T2-A	2019/12/21	4 y	M	Bronchitis	TS	No
24	Y11-62	2c	-		T2-A	2019/12/23	6 y	F	Bronchitis	TS	No
25	Y11-64	2j	-		T2-B	2019/12/23	6 y	M	Bronchitis	TS	No
26	Y11-65	2c	-		T2-A	2020/1/4	8 y	M	Pneumonia	TS	No
27	Y11-66	2j	-		T2-B	2020/1/4	10 y	M	Bronchitis	TS	No
28	Y11-69	2c	-		T2-A	2020/1/6	7 y	M	Bronchitis	TS	No
29	Y11-71	2c	-		T2-A	2020/1/8	6 y	M	Pneumonia	TS	No
30	Y11-72	2j	-		T2-B	2020/1/8	28 y	F	Bronchitis	TS	No
31	Y11-73	2c	-		T2-A	2020/1/9	8 y	M	Bronchitis	TS	No
32	Y11-74	2c	-		T2-A	2020/1/16	6 y	M	Bronchitis	TS	No
33	Y11-75	2c	-		T2-A	2020/1/16	4 y	M	Bronchitis	TS	No
34	Y12-3	2c	-		T2-A	2020/1/18	30 y	F	Bronchitis	TS	No
35	Y12-4	1	-	3	T1-2	2020/1/24	8 y	F	Bronchitis	TS	No
36	Y12-6	2c	-		T2-A	2020/1/27	6 y	M	Bronchitis	TS	No
37	Y12-9	2b2	A2063G		T2-B	2020/1/29	6 y	M	Pneumonia	TS	No
38	Y12-24	2b2	A2063G		T2-B	2020/2/12	13 y	M	Pneumonia	TS	No
39	Y12-28	2c	-		T2-A	2020/2/18	2 y	F	Pneumonia	TS	No
40	Y12-29	2c	-		T2-A	2020/2/18	1 y	F	Pneumonia	TS	No
41	Y12-30	2c	-		T2-A	2020/2/18	11 y	M	Bronchitis	TS	No
42	Y12-36	2c	-		T2-A	2020/2/25	32 y	F	Bronchitis	TS	No
43	Y12-38	2j	-		T2-B	2020/3/7	7 y	F	Pneumonia	TS	No
44	Y12-39	2j	-		T2-B	2020/3/9	8 y	M	Pneumonia	TS	No
45	Y12-42	2j	-		T2-B	2020/3/26	10 y	M	Bronchitis	TS	No

Number of swab specimen : 138
Culture positive rate : 33 % (45/138)

Specimen
TS : Throat swab
